# Monitoring the Activation of Open Metal Sites in [Fe*_x_*M_3–*x*_(μ_3_-O)] Cluster-Based Metal–Organic Frameworks by Single-Crystal X-ray Diffraction

**DOI:** 10.1021/jacs.2c13299

**Published:** 2023-02-15

**Authors:** Wenmiao Chen, Zhi Wang, Qi Wang, Khaoula El-Yanboui, Kui Tan, Heather M. Barkholtz, Di-Jia Liu, Peiyu Cai, Liang Feng, Youcong Li, Jun-Sheng Qin, Shuai Yuan, Di Sun, Hong-Cai Zhou

**Affiliations:** † State Key Laboratory of Coordination Chemistry, School of Chemistry and Chemical Engineering, Nanjing University, Nanjing 210093, P. R. China; ‡ Department of Chemistry, Texas A&M University, College Station, Texas 77843-3255, United States; § School of Chemistry and Chemical Engineering, Shandong University, Jinan 250100, P. R. China; ∥ Department of Materials Science & Engineering, University of Texas at Dallas, Richardson, Texas 75080, United States; ⊥ Chemical Sciences & Engineering Division, Argonne National Laboratory, Lemont, Illinois 60439, United States; # Department of Materials Science and Engineering, Texas A&M University, College Station, Texas 77842, United States

## Abstract

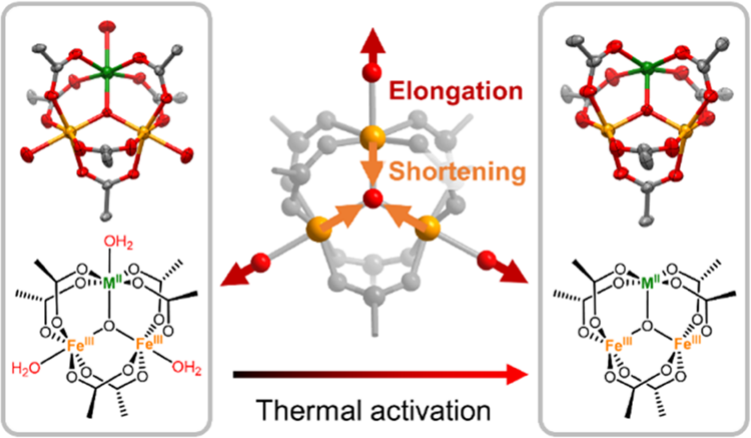

While trinuclear [Fe*_x_*M_3–*x*_(μ_3_-O)] cluster-based metal–organic frameworks (MOFs) have found wide applications in gas storage and catalysis, it is still challenging to identify the structure of open metal sites obtained through proper activations and understand their influence on the adsorption and catalytic properties. Herein, we use in situ variable-temperature single-crystal X-ray diffraction to monitor the structural evolution of [Fe*_x_*M_3–*x*_(μ_3_-O)]-based MOFs (PCN-250, M = Ni^2+^, Co^2+^, Zn^2+^, Mg^2+^) upon thermal activation and provide the snapshots of metal sites at different temperatures. The exposure of open Fe^3+^ sites was observed along with the transformation of Fe^3+^ coordination geometries from octahedron to square pyramid. Furthermore, the effect of divalent metals in heterometallic PCN-250 was studied for the purpose of reducing the activation temperature and increasing the number of open metal sites. The metal site structures were corroborated by X-ray absorption and infrared spectroscopy. These results will not only guide the pretreatment of [Fe*_x_*M_3–*x*_(μ_3_-O)]-based MOFs but also corroborate spectral and computational studies on these materials.

## Introduction

Metal–organic frameworks (MOFs), also known as porous coordination polymers, are a class of crystalline materials constructed from inorganic metal nodes and organic linkers.^1−3^ Because of their large surface area, diverse structures, and tunable functionalities, MOFs have attracted research interest in the fields of gas storage, separation,^4,5^ water harvesting,^6^ catalysis,^7,8^ sensing,^9^ and biomedicine.^10,11^ A variety of metal cations or clusters have been adopted as building units, giving rise to an unlimited number of framework structures.^12^ Among them, MOFs composed of trinuclear [M_3_(μ_3_-O)] (M = Fe^3+^, Cr^3+^, Al^3+^, Ga^3+^, In^3+^, etc.) clusters are one of the most attractive classes of materials owing to their superior chemical stability and structural tunability.^13−16^ Representative examples are the MIL series (MIL-100,^17^ MIL-101,^18^ MIL-88^19,20^), which have found wide applications in gas storage,^21^ separation,^22^ water adsorption,^23^ catalysis,^24^ and drug delivery.^25^ Taking the [Fe_3_(μ_3_-O)] node as an example, each Fe^3+^ is coordinated with four carboxylate O, a (μ_3_-O), and a terminal ligand.^26^ Two Fe^3+^ within the trinuclear cluster are coordinated to solvent molecules, while the remaining is terminated by a counterion. The properties of [Fe_3_(μ_3_-O)]-based MOFs can be further tuned by replacing one of three Fe with heterometals.^27,28^ For example, our group has developed a general method to synthesize heterometallic MOFs based on [Fe_2_M(μ_3_-O)] (M = Fe^3+^, Co^2+^, Ni^2+^, Mn^2+^, Zn^2+^) clusters and identified PCN-250(FeCo) (formerly named soc-MOF^29^ or MIL-127^30^) as a promising H_2_ and CH_4_ adsorbent.^27^ The chemistry of trinuclear [M_3_(μ_3_-O)]-MOFs was further expanded by Feng and co-workers, who reported heterometallic MOFs (CPM-200 series) with both trivalent metals (In^3+^, Ga^3+^, Fe^3+^, V^3+^, Sc^3+^) and divalent metals (Mg^2+^, Mn^2+^, Co^2+^, Ni^2+^).^31^ By screening the combination of different trivalent and divalent metals, CPM-200(FeMg) was found to exhibit the highest CO_2_/N_2_ selectivity.

Coordinatively unsaturated metal sites (or open metal sites) of [Fe_3_(μ_3_-O)] clusters play an important role in catalysis^24^ and gas adsorption.^26^ The exposed metal centers act as active sites to promote catalytic reactions^24^ or regulate the binding of reactants.^32^ Moreover, open metal sites have been demonstrated to enhance the binding affinity of gas adsorbates, including H_2_, CO_2_, SO_2_, CO, NO, C_2_H_2_, and CH_4_.^33^ On the other hand, terminal aqua ligands in PCN-250(Fe) have been demonstrated to benefit to CO_2_ adsorption.^34^ Therefore, it is important to determine the number of open metal sites/coordinating solvents to optimize MOF activation conditions for targeted applications. Researchers have utilized a series of spectroscopic and computational methods to derive insight into the structure of Fe sites during thermal activation, including infrared spectroscopy (IR),^35^^57^Fe Mössbauer,^26^ adsorption microcalorimetry,^26^ X-ray absorption spectroscopy (XAS),^36^ and density functional theory (DFT) simulations.^37^ For example, Férey, Serre, and co-workers have used IR^35^ and Mössbauer^26^ to demonstrate that heating [Fe_3_(μ_3_-O)]-based MIL-101 removes the terminal −H_2_O ligands to expose two Fe^3+^ sites at 150 °C. Further increasing the temperature up to 230 °C removes the −OH anions associated with the reduction of Fe^3+^ to Fe^2+^. Our recent studies have shown that the Fe^3+^ reduction in PCN-250(Fe) is related to the decarboxylation of linkers during high-temperature activation (240 °C, 12 h).^38,39^ However, to our knowledge, direct structural evidence has not been provided by single-crystal X-ray diffraction (SCXRD) to reveal the structural change of Fe^3+^ sites during activation.

In situ SCXRD is a powerful technique to provide critical insight into the structural change of crystalline materials. It has been applied to study the thermal activation and guest–host interactions of divalent metal-based MOFs such as MOF-74(Mg^2+^, Co^2+^, Ni^2+^),^40,41^ UTSA-74(Zn^2+^),^42^ and MFM-170(Cu).^43^ Unfortunately, in situ SCXRD has not been applied to evidence the open Fe^3+^ sites in [Fe_3_(μ_3_-O)]-based MOFs. This is because in situ SCXRD requires sizeable and high-quality single crystals, whereas many Fe–MOFs are obtained as microcrystalline powders. Single-crystalline PCN-250 provides a suitable platform for in situ SCXRD studies. In this study, we used in situ variable-temperature SCXRD to snapshot the structure of PCN-250 during thermal activation. The gradual exposure of two Fe^3+^ sites was observed as the temperature increased from 323 to 473 K, which was accompanied by the elongation of terminal Fe–OH/H_2_O bonds and shortening of Fe-μ_3_-O bonds. In addition, partially replacing Fe^3+^ with divalent metals (M = Ni^2+^, Co^2+^, Zn^2+^, Mg^2+^) was shown to reduce the activation temperature and increase the number of open metal sites while the effects of different metals on thermal activation were compared. The structural changes observed by SCXRD were further corroborated by XAS and IR studies. This in situ SCXRD study will complement and validate the spectroscopic and computational efforts on the activation of Fe-based MOFs with open metal sites.

## Results and Discussion

### Synthesis of PCN-250(FeM)

**Figure 1:**
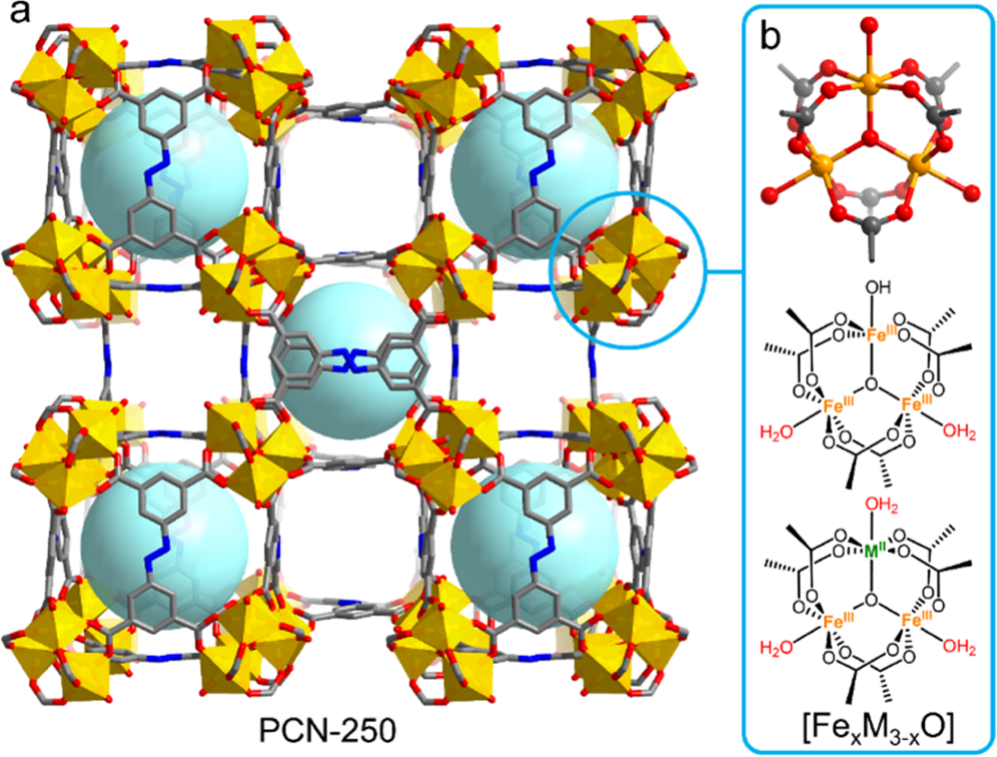
Structure of PCN-250 and the [Fe_2_M(μ_3_-O)] clusters (M = Ni^2+^, Co^2+^, Zn^2+^, Mg^2+^). Light blue spheres indicate the cubic cages. Color scheme: metals, orange; O, red; carbon, black; N, blue.

Single crystals of PCN-250 were synthesized by the solvothermal reaction of Fe(NO_3_)_3_·9H_2_O (or a mixture of Fe(NO_3_)_3_ 9H_2_O and divalent metal salts) and 3,3′,5,5′-azobenzenetetracarboxylic acid (H_4_ABTC) in DMF using acetic acid as a modulator at 150 °C. They are isostructural MOFs with similar PXRD patterns (Figure S1) that all match the simulation based on the crystal structure of PCN-250(Fe). Within the crystal structure of PCN-250, three metal cations in the octahedral coordination environment are bridged by μ_3_-O to form a [Fe*_x_*M_3–*x*_(μ_3_-O)] core, which is further linked by six carboxylate groups from ABTC ligands into a **soc** network (Figure 1a). The ratio of divalent metal is defined as the moles of divalent metal divided by the total moles of metals, which corresponds to (3 – *x*)/3 in the chemical formula of [Fe*_x_*M_3–*x*_(μ_3_-O)]. Based on the inductively coupled plasma mass spectrometry (ICP-MS), the ratios of divalent metals were determined to be 0.298, 0.263, 0.286, and 0.303 for PCN-250(Fe_2_Mg), PCN-250(Fe_2_Zn), PCN-250(Fe_2_Co), and PCN-250(Fe_2_Ni), which approximate a cluster composition of [Fe_2_M(μ_3_-O)]. Furthermore, the Ni ratio within the material can be enhanced to 0.663 by increasing the Ni content in the precursor during MOF synthesis, giving rise to PCN-250(FeNi_2_). EDX-SEM elemental mapping reveals that the metal ions are uniformly distributed within the MOF crystals (Figure S2). The heterometallic samples show BET surface area and N_2_ total uptake comparable to that of PCN-250(Fe), in agreement with their similar framework structures (Figure S3). The slightly higher gravimetric BET surface area and N_2_ total uptake of PCN-250(Fe_2_Mg) can be attributed to the lower atomic weight of Mg. Overall, the PXRD, ICP-MS, EDX, and N_2_ adsorption measurements confirm the successful synthesis of PCN-250(FeM) (M = Fe^3+^, Ni^2+^, Co^2+^, Zn^2+^, Mg^2+^).

Based on the literature,^26,37^ three terminal ligands on the trinuclear cluster can be either solvent molecules (e.g., H_2_O) or counter anions (e.g., OH^–^, F^–^) to keep neutrality. Therefore, the trinuclear cluster can be formulated as [Fe_3_(μ_3_-O)(COO)_6_(X)(S)_2_] or [Fe_2_M(μ_3_-O)(COO)_6_(S)_3_] (X = counter anions, S = solvents, M = Ni^2+^, Co^2+^, Zn^2+^, Mg^2+^, Figure 1b). We assume the terminal counterions/ligands are −OH/(H_2_O)_2_ in PCN-250(Fe) and −(H_2_O)_3_ in PCN-250(FeM). The existence of terminal −OH/H_2_O was verified by IR spectra after removing methanol solvents in the pores under vacuum (Figure S4). Both IR spectra of PCN-250(Fe) show the ν(OH) band at 3734 and 3680 cm^–1^, corresponding to the terminal −OH and −H_2_O, respectively.^44^ The relative intensity terminal −OH band in PCN-250(Fe_2_Ni) is much lower, since the open metal sites are predominately terminated by −(H_2_O)_3_ in PCN-250(FeM). Single-crystal structures of PCN-250(Fe) and PCN-250(FeM) indicate that the terminal sites were occupied by O atoms, whereas methanol molecules cannot be refined. Nevertheless, we cannot rule out the possibility that methanol molecules partially occupy the terminal sites, since the methyl group from methanol can be disordered and cannot be determined by SCXRD.

### Variable-Temperature SCXRD of PCN-250(Fe)

**Figure 2:**
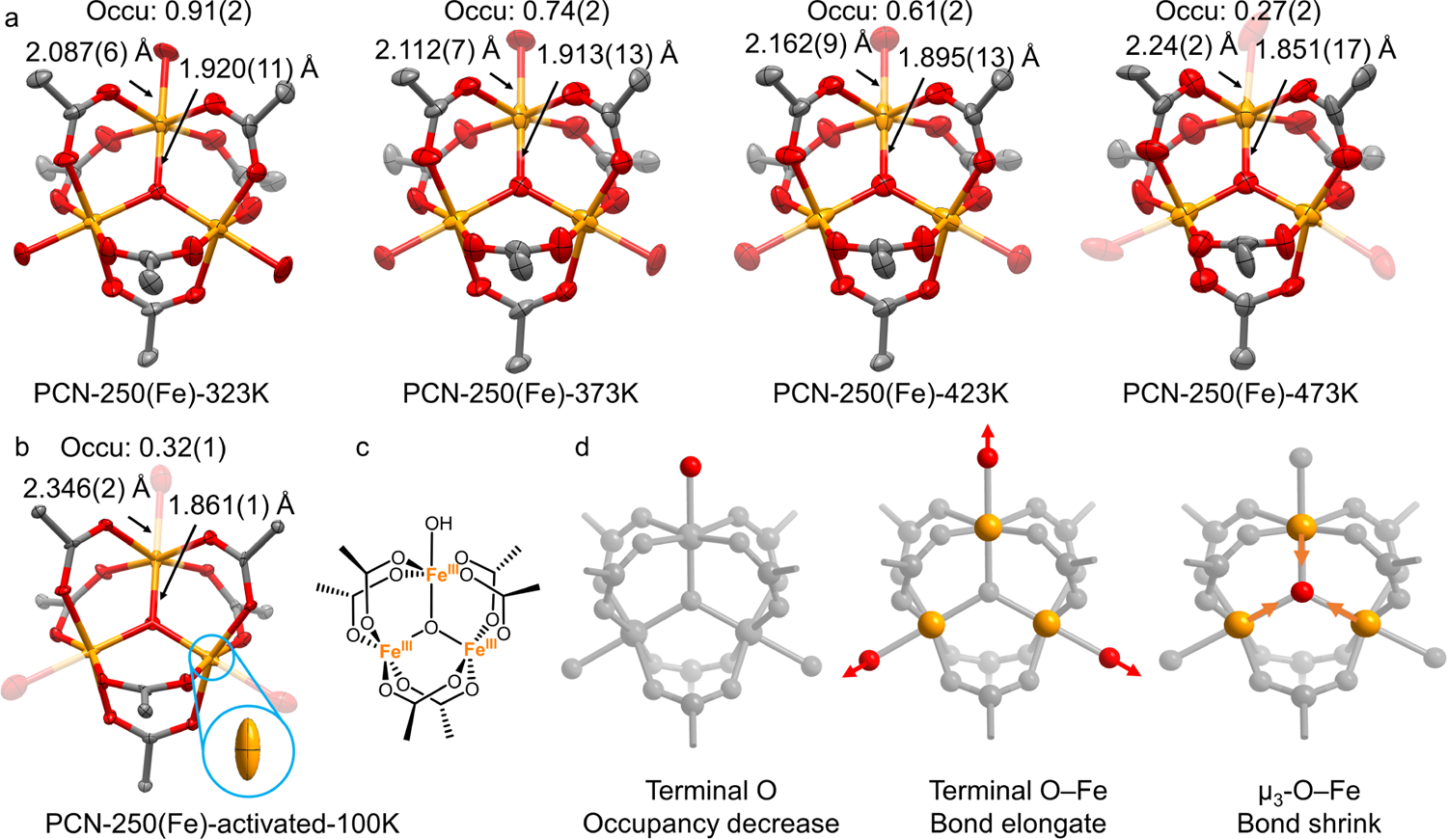
In situ variable-temperature SCXRD analysis of PCN-250(Fe). Single-crystal structures of the [Fe_3_(μ_3_-O)] cluster at (a) 323, 373, 423, and 473 K. (b) Single-crystal structures of the [Fe_3_(μ_3_-O)] cluster at 100 K after thermal activation (473 K for 1 h). The inset highlights the anisotropy of Fe atoms in activated PCN-250(Fe) at 100 K. The structures are shown as the ellipsoid model with 50% probability. (c) Proposed molecular structure of the [Fe_3_(μ_3_-O)] cluster after thermal activation. (d) Schematic representation of the structure change during thermal activation for PCN-250(Fe), including the decreasing of terminal O occupancy, elongation of terminal O–Fe bonds, and shrinkage of μ_3_-O–Fe bonds.

A single crystal of PCN-250(Fe) was placed under N_2_ flow at different temperatures for 1 h to represent the thermal activation process. Consecutive SCXRD data were collected using the same crystal at 323, 373, 423, and 473 K. Single-crystal structures of PCN-250(Fe) at different temperatures show identical frameworks and similar lattice parameters (Table S1). However, a close inspection of Fe sites reveals a significant change in the Fe coordination environments (Figure 2a). Three Fe atoms within the [Fe_3_(μ_3_-O)] clusters are crystallographically identical, and the terminal −H_2_O/OH ligands are substitutionally disordered. To determine the number of terminal ligands on the Fe sites, the site occupancy factors of terminal O were refined freely. It has been observed that the occupancy of terminal O atoms decreases from 0.91 to 0.27, with a concomitant increase in terminal O–Fe bonds (from 2.087 to 2.24 Å) and shortening of μ_3_-O–Fe bonds (from 1.920 to 1.851 Å) from 323 to 473 K, respectively. These results indicate that the thermal treatment weakens the terminal O–Fe bonds and induces the removal of terminal −H_2_O molecules (Figure 2d). As a result, the coordination geometry of Fe transformed from an octahedron to a square pyramid, where the axial bonds (μ_3_-O–Fe, 1.857 Å) were shorter than the equatorial bonds (COO–Fe, 1.994 Å). The reduced symmetry of the Fe coordination sphere (from octahedron to square pyramid) is also reflected by the color change of PCN-250(Fe) crystals from dark red to black (Figure S5).

To collect high-quality single-crystal data of thermally activated PCN-250(Fe) (473 K for 1 h), the temperature was further cooled to 100 K. The terminal O occupancy and bond lengths were not significantly changed upon cooling (Figure 2b), suggesting that the aforementioned structural change was not attributed to temperature factors. Compared to the as-synthesized PCN-250(Fe), the high anisotropic displacement parameter of Fe (*U*_11_ = 0.022), as reflected by the ellipse model (Figure 2b inset), suggests that two types of Fe sites (octahedral and square pyramidal geometries) coexist in the structure. The slightly higher equivalent isotropic displacement parameter (*U*_eq_ = 0.051) of terminal O atoms might be attributed to the impact of N_2_ molecules around the open Fe^3+^ sites. Based on the crystal structure, the clusters after thermal activation can be formulated as [Fe_3_(μ_3_-O)(COO)_6_(OH)], where two terminal −H_2_O molecules were removed, leaving a −OH to balance the charge (Figure 2c). The occupancy of the remaining −OH (0.27) is slightly lower than the formula (0.33), which can be explained by the partial removal of −OH with a concomitant Fe^3+^ reduction to Fe^2+^. This thermally induced Fe^3+^ reduction has been previously observed in [Fe_3_(μ_3_-O)]-based MOFs, including MIL-101 (Fe)^26^ and PCN-250(Fe).^38^

To eliminate the effect of thermal treatment time on the number of open Fe^3+^ sites, the single crystal of PCN-250(Fe) was heated at 473 K for 1, 2, and 4 h before SCXRD analysis (Figure S6 and Table S2), which did not cause a significant change in terminal O occupancy (0.31, 0.30, and 0.30). Therefore, the equilibrium time in our measurements (1 h) is sufficient for H_2_O molecules to escape from the MOF crystals. Further increasing the temperature above 473 K caused degradation of the diffraction quality for PCN-250(Fe) crystals. Meanwhile, variable-temperature PXRD of PCN-250(Fe) in N_2_ atmosphere indicates that the framework maintained its crystallinity from 323 to 573 K, beyond which the diffraction peaks started to decrease (Figure S7). Similarly, thermogravimetric analysis (TGA) did not show framework decomposition until 573 K (Figure S8). The degradation of single-crystal diffraction quality was tentatively attributed to the decarboxylation of ABTC linkers at temperatures above 473 K, which has been documented in the literature.^38^ The decarboxylation of ABTC linkers induced defects in PCN-250(Fe), which disrupted the local structures while maintaining the long-range order. Therefore, the single-crystal diffraction quality was significantly reduced, but the PXRD patterns remained unchanged. As a result, the structure of PCN-250(Fe) above 473 K cannot be determined by SCXRD, which requires future studies by in situ synchrotron PXRD or neutron powder diffraction.

The cycling stability of PCN-250(Fe) toward solvation and reactivation was confirmed by the SCXRD (Figure S9 and Table S3). Exposing activated PCN-250(Fe) in the air at room temperature for 12 h leads to the reoccupation of terminal ligands (possibly, water from the air). The resulting PCN-250(Fe) sample can be reactivated by heating, as confirmed by the partial removal of terminal ligands in the crystal structure. However, the occupancy of terminal ligands in the second-round activation is lower than that of the first-round activation, indicating that the open metal sites are not fully reoccupied by water within 12 h in air. Interestingly, the occupancy of the terminal −OH is reduced to 0.20 at 473 K, which is lower than that in first-round activation (0.27 at 473 K). Upon cooling the reactivated sample to 100 K, we observed the binding of N_2_ to the open metal sites with an occupancy of 0.43, which can be explained by the stronger binding N_2_ to the low-valent Fe^II^. These results are consistent with the previous literature that observed the removal of the terminal −OH accompanied by the reduction of Fe^III^ to Fe^II^ during the long-time activation of Fe-based MOFs.^26,38^

### Effect of Ni^2+^ Substitution on Thermal Activation

**Figure 3:**
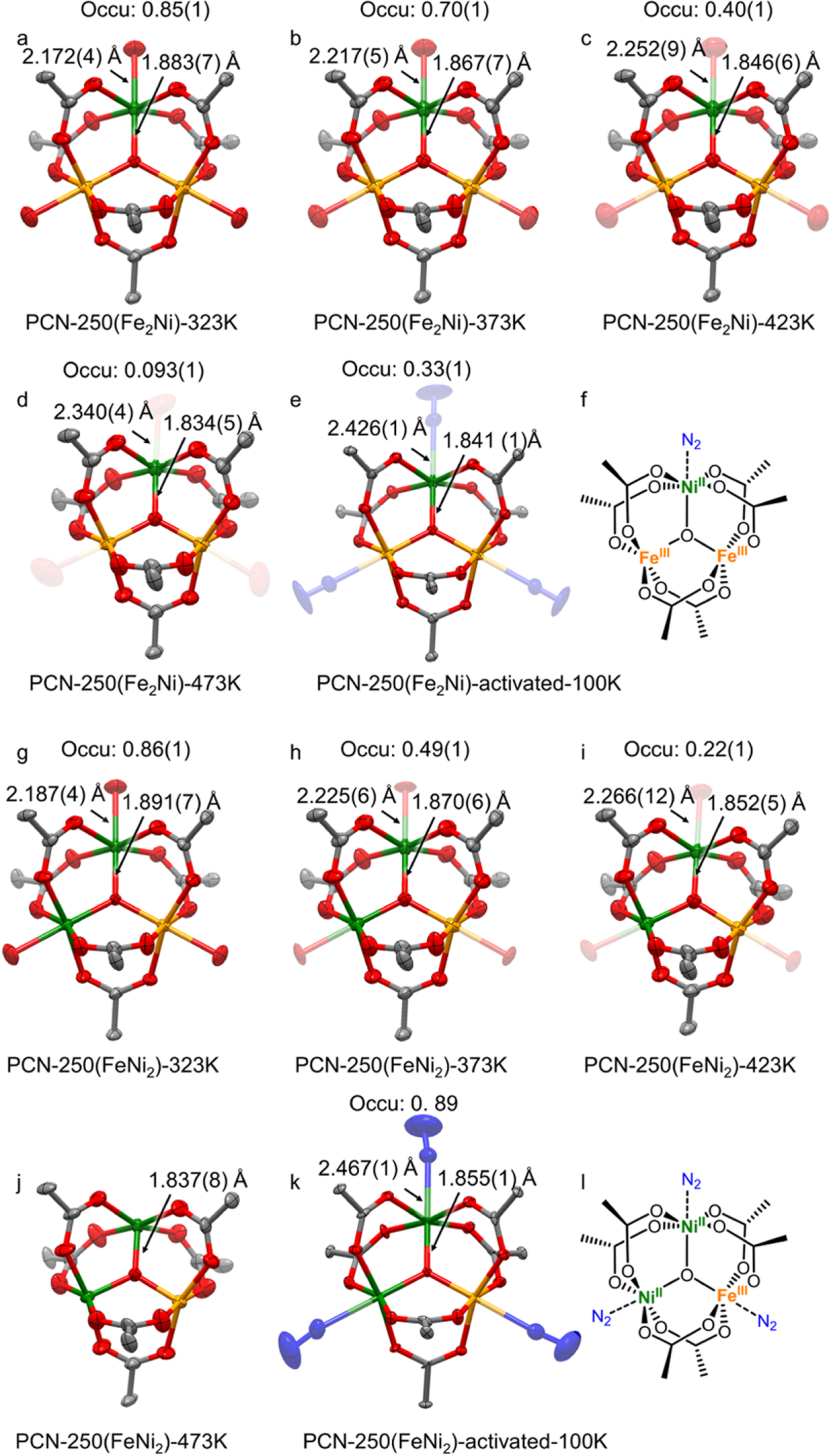
In situ variable-temperature SCXRD analysis of PCN-250(Fe_2_Ni) and PCN-250(FeNi_2_). Single-crystal structures of PCN-250(Fe_2_Ni) showing the [Fe_2_Ni(μ_3_-O)] cluster at 323 K (a), 373 K (b), 423 K (c), 473 K (d), and 100 K after thermal activation at 473 K for 3 h (e) using the ellipsoid model (50% probability). (f) Proposed molecular structure of the [Fe_2_Ni(μ_3_-O)] cluster at 100 K after thermal activation at 473 K for 3 h. Single-crystal structures of PCN-250(FeNi_2_) showing the [FeNi_2_(μ_3_-O)] cluster at 323 K (g), 373 K (h), 423 K (i), 473 K (j), and 100 K after thermal activation at 473 K for 3 h (k) using the ellipsoid model (50% probability). (l) Proposed molecular structure of the [FeNi_2_(μ_3_-O)] cluster at 100 K after thermal activation at 473 K for 3 h.

We further investigated the effects of divalent metal ion substitution on the thermal activation process of PCN-250 using variable-temperature SCXRD. Since the Fe and doped divalent metal atoms are substitutionally disordered, precise refinement and identification of single atoms are limited using SCXRD. It has been reported that partially replacing Fe^3+^ with divalent metals (Ni^2+^, Co^2+^, Mg^2+^) reduced the activation temperature and exposed more metal sites.^28^ Similar behaviors were observed for PCN-250(Fe_2_Ni) and PCN-250(FeNi_2_) by variable-temperature SCXRD (Tables S4 and S5). Generally, the trend of structural change for PCN-250(Fe_2_Ni) and PCN-250(FeNi_2_) is similar to that of PCN-250(Fe), which all involve a decrease in terminal O occupancy, elongation of terminal O–Fe bonds, and shortening of μ_3_-O–Fe bonds. By incorporating one Ni per cluster, the occupancy of terminal O was substantially reduced compared to that of PCN-250(Fe), especially at elevated temperatures (Figure 3a–d). Specifically, the terminal O occupancy of PCN-250(Fe_2_Ni) was reduced to 0.093 at 473 K (Figure 3d), which is significantly smaller than that of PCN-250(Fe) (0.27). This can be explained by the fact that low-valent Ni^2+^ substitution caused the terminal ligand change from [Fe_3_^3+^(μ_3_-O)]–(OH)/(H_2_O)_2_ to [Fe_2_^3+^Ni^2+^(μ_3_-O)]–(H_2_O)_3_, where all terminal −H_2_O can be removed at 473 K. The remaining terminal O might imply the existence of some [Fe_3_^3+^(μ_3_-O)]–(OH)/(H_2_O)_2_ clusters in PCN-250(Fe_2_Ni), possibly due to the nonuniform distribution of Ni among clusters. Interestingly, when cooling down the activated PCN-250(Fe_2_Ni) under N_2_ flow at 100 K, the binding of N_2_ molecules to the metal sites was observed with the N_2_ occupancy of 0.33 (Figure 3e). The N≡N bond distance was refined to be 1.081 Å, corresponding to a triple bond. Meanwhile, the M···N distance of 2.426 Å suggests weak metal···N_2_ interactions. We propose that N_2_ binds to the Ni sites, which corresponds to the N_2_ occupancy of 0.33 (Figure 3f). It is known that the end-on coordinate nitrogen interacts with the octahedral metal ion through d_*z*_^2^ orbital and results in a three-center σ bond with two electrons each in a bonding and nonbonding orbital. Furthermore, the unoccupied π* orbitals of N_2_ can accept backbonding electrons from the metal dπ orbitals. Ni(II) with d^8^ configuration has more dπ electrons than Fe(III) with d^5^ configuration, resulting in stronger π backbonding in Ni-substituted PCN-250. A similar observation has been documented where Ni doping in Fe–MOFs increases the adsorption of CO and NO by π-backbonding.^28^ It should be noted that the remaining terminal −OH still exists at 100 K. However, the low occupancy of −OH (0.093) and the overlap with the N_2_ electrons make it difficult to independently refine −OH and −N_2_.

By increasing the Ni ratios, PCN-250(FeNi_2_) shows further reduced terminal O occupancy than PCN-250(Fe) and PCN-250(Fe_2_Ni) (Figure 3g–j). The terminal O occupancy decreased to 0.22 at only 423 K, which is even lower than that of PCN-250(Fe) at 473 K (Figure 3i). More importantly, the terminal O ligands of PCN-250(FeNi_2_) were completely removed at 473 K (Figure 3j), as indicated by the absence of refinable diffraction peaks around metal sites. When cooling down the activated PCN-250(FeNi_2_) to 100 K, the occupancy of N_2_ was 0.89 (Figure 3k), suggesting that N_2_ molecules may bind to both Ni^2+^ and Fe^3+^ sites (Figure 3l). In contrast, PCN-250(Fe) has a neglectable N_2_ binding, although two Fe^3+^ sites are exposed. This phenomenon can be attributed to the electronic modulation of Fe^3+^ sites by neighboring Ni^2+^, which has been demonstrated to change the binding energy of molecules.^45^ The DFT-calculated adsorption energy of N_2_ increases with the Ni content (33, 41, and 42 kJ mol^–1^ for Fe_3_, Fe_2_Ni, and FeNi_2_ clusters, DFT calculation results in Table S6, details in Supporting Information), which is in agreement with the higher occupancy of N_2_ in Ni-substituted PCN-250(Fe_2_Ni) and PCN-250(FeNi_2_).

The effect of Ni substitution on reducing the MOF activation temperature was further studied by N_2_ adsorption isotherms (Figure S10). PCN-250(Fe) activated at low temperature (denoted as PCN-250(Fe)-423 K) shows a 13% lower BET surface area than the high-temperature activated PCN-250(Fe)-473 K. On the other hand, the BET surface area difference (5%) between PCN-250(Fe_2_Ni)-423 K and PCN-250(Fe_2_Ni)-473 K was less prominent. In addition, the PCN-250(Fe_2_Ni) samples show a higher BET surface area and N_2_ uptake than PCN-250(Fe) activated at the same temperature, indicating that Ni substitution facilitates the removal of terminal O ligands and reduces the activation temperature.

### Effect of Different Divalent Metals on Thermal Activation

**Figure 4:**
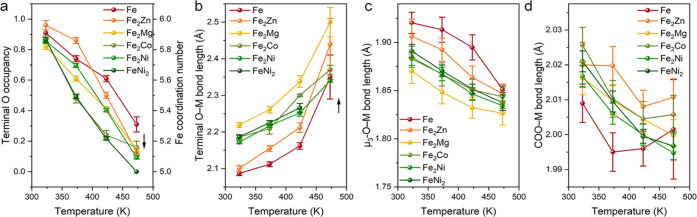
Comparison of terminal O occupancy (a), terminal O–M bond lengths (b), μ_3_-O–M bond lengths (c), and COO–M bond lengths (d) determined by variable-temperature SCXRD for PCN-250(Fe), PCN-250(Fe_2_Mg), PCN-250(Fe_2_Zn), PCN-250(Fe_2_Co), PCN-250(Fe_2_Ni), and PCN-250(FeNi_2_).

We further conducted variable-temperature SCXRD analysis on other divalent metal-substituted PCN-250, including PCN-250(Fe_2_Mg), PCN-250(Fe_2_Zn), and PCN-250(Fe_2_Co) (Tables S7–S9). They all show a similar thermally induced decrease in terminal O occupancy, elongation of terminal O–M bonds, and shortening of μ_3_-O–M bonds. The terminal O occupancy and O–Fe bond lengths under different temperatures have been compared (Figure 4). Although the initial terminal O occupancy in all of the samples (323 K) is comparable, divalent metal-substituted PCN-250 shows a more pronounced O occupancy decrease as the temperature increases (Figure 4a). As a result, the terminal O occupancy at 473 K in heterometallic samples is much lower than that of pure Fe-based PCN-250(Fe). On the other hand, the difference of PCN-250(Fe_2_M) (M = Ni^2+^, Co^2+^, Zn^2+^, Mg^2+^) in terminal O occupancy at 473 K is not significant, as reflected by the largely overlapped error bars. Meanwhile, the incorporation of divalent metals induced a noticeable elongation of terminal O–M bonds (Figure 4b) and shortening of μ_3_-O–M bonds (Figure 4c), indicating a more distorted octahedral coordination geometry. Generally, the terminal O–M bond length follows the trend of Mg > Co–Ni > Zn (μ_3_-O–M bond length Mg < Co–Ni < Zn),^46^ which is consistent with previous reports of mixed-metal CPM-200s and PCN-250.^31,39^ This observation is consistent with the trend that terminal O ligands are more likely to be removed upon divalent metal incorporation. Compared with the terminal O–M bonds and μ_3_-O–M bonds, the thermal-induced variation of COO–M bonds is less obvious (within 0.02 Å, Figure 4c). This can be explained by the fact that the coordination of equatorial ligands (COO) is not sensitive to the change in axial ligands. The initial decrease in COO–M bonds upon heating is caused by the reduction of metal coordination numbers. The slight increase of COO–M bond lengths at 473 K for PCN-250(Fe), PCN-250(Fe_2_Zn), and PCN-250(Fe_2_Co), can be attributed to the temperature-induced bond elongation. The average COO–M bond length of PCN-250(Fe) is shorter than that of the heterometallic samples, in agreement with the smaller ionic radius of Fe^3+^ than divalent M^2+^.^47^ Overall, these results imply that the divalent metals can effectively modulate the coordination environment of [Fe*_x_*M_3–*x*_(μ_3_-O)] bimetallic clusters to facilitate desolvation (i.e., H_2_O removal) during thermal activation.

After thermal activation at 473 K for 1 h, the single crystals of divalent metal-substituted PCN-250(Fe_2_M) (M = Co^2+^, Zn^2+^, Mg^2+^) were further cooled to 100 K to collect high-quality single-crystal data. The bond lengths and terminal O occupancy of activated PCN-250(Fe_2_M) (M = Co^2+^, Zn^2+^, Mg^2+^) at 100 K are not significantly changed compared to the data at 473 K. Although the terminal O occupancy of PCN-250(Fe_2_M) (M = Co^2+^, Zn^2+^, Mg^2+^) is similar to that of PCN-250(Fe_2_Ni), the binding of N_2_ molecules to the open metal sites was not observed in any of these samples, except for Ni^2+^-substituted ones (Figure S11). This result reveals the unique role of Ni^2+^ in forming stronger interactions with N_2_ molecules, possibly through π-backbonding. The mechanism and applications will be studied in our future research.

### XAS and IR Characterizations

**Figure 5:**
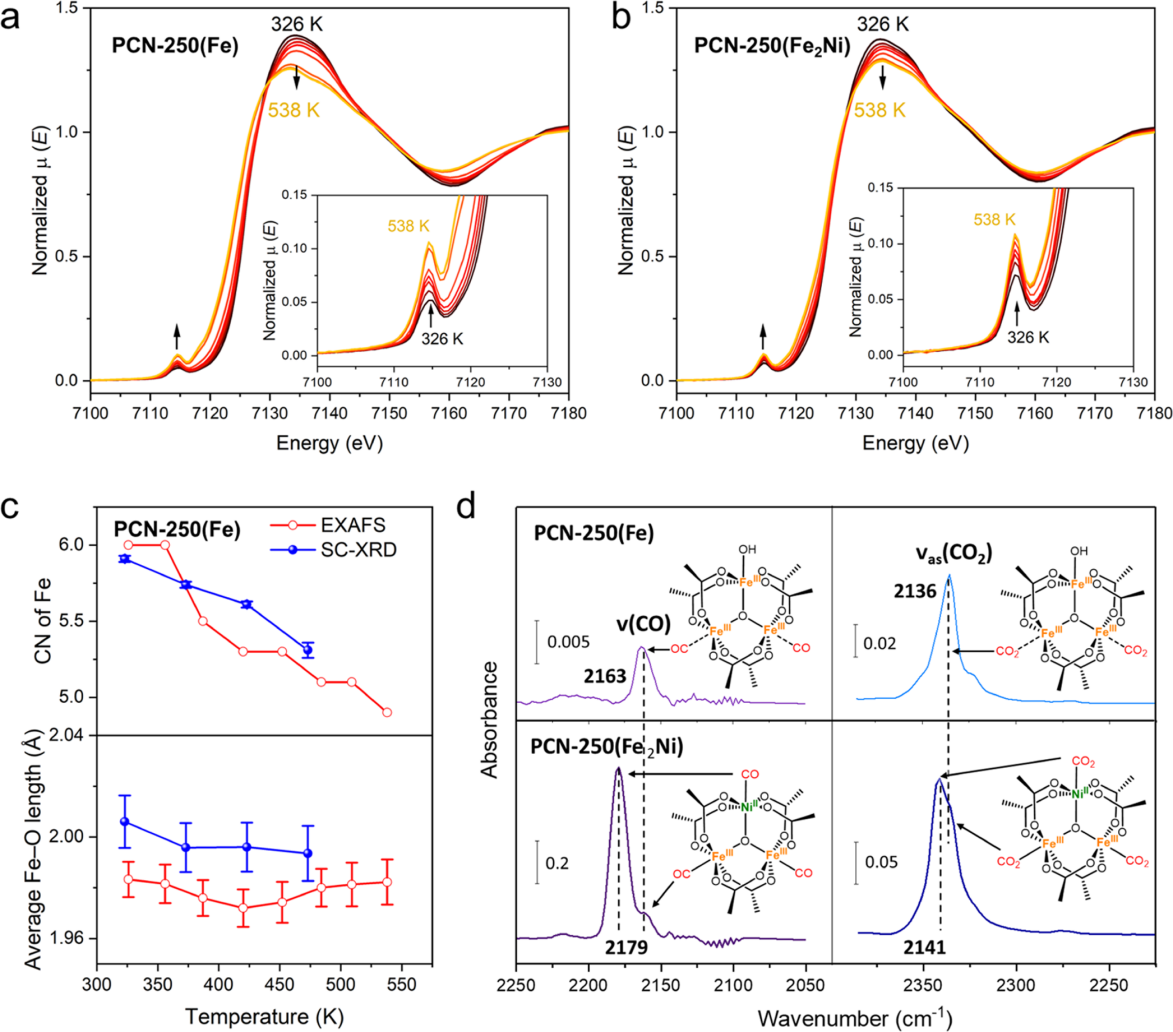
In situ variable-temperature Fe K-edge XANES characterization of (a) PCN-250(Fe) and (b) PCN-250(Fe_2_Ni) from 326 to 538 K. Insets highlight the pre-edge peaks. (c) Coordination numbers (CN) and average Fe–O bond lengths from EXAFS fitting and single-crystal structures under different temperatures. (d) IR spectra probing open metal sites in PCN-250(Fe) and PCN-250(Fe_2_Ni) using CO (∼40 torr) and CO_2_ molecules (∼6 torr). The stretching bands ν(CO) and ν(CO_2_) of adsorbed species are presented, and the gas-phase signal of both CO and CO_2_ was subtracted. The lower dosing pressure of CO_2_ is selected since IR signal of the gas-phase CO_2_ is prohibitively high above 10 Torr, making the observation of adsorbed CO_2_ impossible.

The single-crystal structures were further correlated to the XAS and IR spectra. In situ variable-temperature Fe K-edge XANES spectra of PCN-250(Fe_2_Ni) were collected from 326 to 538 K (Figure 5b). Our previously reported XANES spectra of PCN-250(Fe)^39^ were further analyzed for comparison (Figure 5a). The X-ray absorption near edge structure (XANES) spectra of PCN-250(Fe) and PCN-250(Fe_2_Ni) taken at Fe K-edge show the main absorption peaks at 7134 eV, known as the “white line” with its intensity corresponding to the oxidation state of the octahedral Fe^3+^ species (Figure 5a,b). Upon heating, the main peak at 7134 eV decreases, which can be attributed to the loss of the electron-withdrawing oxygen ligand (from 6 to 5) and therefore higher electronic density over Fe, as it appears to be reduced from Fe^3+^. Meanwhile, the pre-edge peak at 7114 eV increases, which is in agreement with the reduced centrosymmetry of the Fe coordination sphere (from octahedron to square pyramid). This result is in line with previous XAS studies on MIL-100(Fe).^36^ While the main peak of PCN-250(Fe_2_Ni) gradually changes with temperature, the PCN-250(Fe) sample shows a dramatic peak drop at 484 K, indicating a significant change in Fe electronic and coordination structures. Based on the literature,^35^ this structural change at 484 K was tentatively attributed to the reduction of Fe^3+^ to Fe^2+^, accompanied by −OH removal. The extended X-ray absorption fine structure (EXAFS) analysis shows a dominant Fe–O peak in the spectra taken at different temperatures when the samples were heated in He flow (Figure S12), and the reduction of Fe–O peak intensity at 484 K was observed, in agreement with the removal of the terminal −H_2_O. The Fe coordination number and Fe–O distances were further calculated by EXAFS fitting (Table S10), which correlates to the single-crystal data (Figure 5c). The existence of exposed metal sites was further confirmed by infrared spectroscopy using CO and CO_2_ as probe molecules, the vibrational frequency of which show great sensitivity to the metal local environment.^48−50^ The PCN-250(Fe) and PCN-250(Fe_2_Ni) samples were first activated at 473 K for 1 h under vacuum to remove guest solvents and then cooled back to 298 K to dose with CO and CO_2_, respectively. Upon exposure of the samples to CO, a band at 2163 cm^–1^ is observed in PCN-250(Fe), which is attributed to CO molecules adsorbed at the exposed Fe^3+^ sites based on the assignment established in the literature.^28^ In PCN-250(Fe_2_Ni), a prominent band occurs at 2179 cm^–1^. According to the prior IR study of CO adsorption on Ni–MOF-74 that showed Ni^2+^-bound CO appears at 2178 cm^–1^,^51^ this band at 2179 cm^–1^ in PCN-250(Fe_2_Ni) clearly arises from CO adsorbed onto the exposed Ni^2+^ sites. The band at 2163 cm^–1^, due to Fe^3+^-bound CO, is still noticeable in PCN-250(Fe_2_Ni), confirming the coexistence of exposed Fe^3+^ sites. A similar result is also found in CO_2_ dosing experiment, i.e., a single band at 2336 cm^–1^ is observed in PCN-250(Fe) that is ascribed to adsorbed CO_2_ at the Fe^3+^ sites, and two bands at 2341 and 2336 cm^–1^ are present in PCN-250(Fe_2_Ni) that correspond to adsorbed CO_2_ at the Ni^2+^ and Fe^3+^ sites, respectively.^44^ Generally, PCN-250(Fe_2_Ni) shows higher CO and CO_2_ intensities than PCN-250(Fe), which was attributed to the higher density of exposed metal sites and stronger Ni-gas interactions. Together, the XAS and IR studies complement and validate the single-crystal structures to identify the metal site structures upon thermal activation.

## Conclusions

In conclusion, we have used in situ variable-temperature SCXRD to monitor the structural evolution of [Fe*_x_*M_3–*x*_(μ_3_-O)]-based MOFs under different thermal activation conditions. The exposure of the open Fe^3+^ site has been clearly observed along with the gradual distortion of FeO_6_ octahedron to eventually form FeO_5_ square pyramid. Furthermore, the effect of divalent metals on reducing the activation temperature of [Fe*_x_*M_3–*x*_(μ_3_-O)]-based MOFs has been compared. This work provides direct structural evidence of [Fe*_x_*M_3–*x*_(μ_3_-O)]-based MOFs under different activation conditions. The solid structural models will further facilitate computational efforts in understanding and predicting the properties of MOFs. Considering that the exposure of open metal sites is directly related to the gas adsorption and catalytic properties, this work is expected to guide the pretreatment of Fe-based MOFs for a wide range of applications, including gas adsorption, separation, catalysis, and beyond.

## Experimental Section

### Synthesis of PCN-250(Fe)

PCN-250(Fe) was synthesized following the literature with modifications.^27,29^ A mixture of Fe(NO_3_)_3_·9H_2_O (500 mg, 1.23 mmol), H_4_ABTC (100 mg, 0.28 mmol), acetic acid (10 mL), and DMF (20 mL) was charged in a 40 mL Pyrex vial and heated in an oven at 150 °C for 24 h. After cooling to room temperature, dark red crystals were collected by filtration.

### Synthesis of PCN-250(Fe_2_Ni)

A mixture of Fe(NO_3_)_3_·9H_2_O (300 mg, 0.74 mmol), Ni(NO_3_)_2_·6H_2_O (150 mg, 0.51 mmol), H_4_ABTC (100 mg, 0.28 mmol), acetic acid (10 mL), and DMF (20 mL) was charged in a 40 mL Pyrex vial and heated in an oven at 150 °C for 24 h. After cooling to room temperature, dark red crystals were collected by filtration. The Ni/(Ni + Fe) ratio was determined to be 0.303 based on ICP-MS.

### Synthesis of PCN-250(FeNi_2_)

A mixture of Fe(NO_3_)_3_·9H_2_O (100 mg, 0.24), Ni(NO_3_)_2_·6H_2_O (290 mg, 1.00 mmol), H_4_ABTC (100 mg, 0.28 mmol), acetic acid (10 mL), and DMF (20 mL) was charged in a 40 mL Pyrex vial and heated in an oven at 150 °C for 24 h. After cooling to room temperature, dark red crystals were collected by filtration. The Ni/(Ni + Fe) ratio was determined to be 0.663 based on ICP-MS.

### Synthesis of PCN-250(Fe_2_Co)

A mixture of Fe(NO_3_)_3_·9H_2_O (100 mg, 0.24), Co(NO_3_)_2_·6H_2_O (400 mg, 1.37 mmol), H_4_ABTC (100 mg, 0.28 mmol), acetic acid (10 mL), and DMF (20 mL) was charged in a 40 mL Pyrex vial and heated in an oven at 150 °C for 24 h. After cooling to room temperature, dark red crystals were collected by filtration. The Co/(Co + Fe) ratio was determined to be 0.286 based on ICP-MS.

### Synthesis of PCN-250(Fe_2_Zn)

A mixture of Fe(NO_3_)_3_·9H_2_O (100 mg, 0.24), Zn(NO_3_)_2_·6H_2_O (500 mg, 1.34 mmol), H_4_ABTC (100 mg, 0.28 mmol), acetic acid (10 mL), and DMF (20 mL) was charged in a 40 mL Pyrex vial and heated in an oven at 150 °C for 24 h. After cooling to room temperature, dark red crystals were collected by filtration. The Zn/(Zn + Fe) ratio was determined to be 0.263 based on ICP-MS.

### Synthesis of PCN-250(Fe_2_Mg)

A mixture of Fe(NO_3_)_3_·9H_2_O (100 mg, 0.24), Mg(NO_3_)_2_ 6H_2_O (400 mg, 1.56 mmol), H_4_ABTC (100 mg, 0.28 mmol), acetic acid (10 mL), and DMF (20 mL) was charged in a 40 mL Pyrex vial and heated in an oven at 150 °C for 24 h. After cooling to room temperature, dark red crystals were collected by filtration. The Mg/(Mg + Fe) ratio was determined to be 0.298 based on ICP-MS.

### Variable-Temperature SCXRD

Single crystals of PCN-250(FeM) with similar size (∼150 μm) were chosen under an optical microscope and mounted on a glass fiber for SCXRD data collection. Variable SCXRD data were collected on a Rigaku Oxford Diffraction XtaLAB Synergy-S diffractometer with Cu Kα radiation source (λ = 1.54184 Å) using an Oxford Cryostream-800 to control the temperature. The same single crystal was heated at 323, 373, 423, and 473 K under N_2_ flow for 1 h before data collection. After the data collection at 473 K, the temperature was cooled to 100 K to collect high-quality single-crystal data of the thermally activated sample.

### In Situ Infrared (IR) Spectroscopy

In situ IR measurements were performed on a Nicolet 6700 FTIR spectrometer using a liquid N_2_-cooled mercury cadmium telluride (MCT-A) detector. The spectrometer is equipped with a vacuum cell that is placed in the main compartment with the sample at the focal point of the infrared beam. The samples (∼5 mg) were gently pressed onto a tungsten mesh (∼1 cm diameter, wire diameter: 0.001″; width opening: 0.0090″; open area: 81.0%) and placed into the vacuum cell that is connected to a vacuum line for evacuation. The samples were activated by overnight evacuation at 473 K and then cooled back to room temperature for CO and CO_2_ gas adsorption measurement.

## Supporting Information Available

Experimental details; PXRD, SEM-EDX, IR, N_2_ adsorption measurements, TGA analysis; and SCXRD refinement details (PDF)
