# Supplementary material for: Monitoring the Activation of Open Metal Sites in [FexM3–x(μ3-O)] Cluster-Based Metal–Organic Frameworks by Single-Crystal X-ray Diffraction
Source: J Am Chem Soc. 2023 Feb 15;145(8):4736–45. doi: 10.1021/jacs.2c13299 (PMC10848254; doi:10.1021/jacs.2c13299)
Supplement: Supplementary file 1 — ja2c13299_si_001.pdf [file ja2c13299_si_001.pdf]

Supplementary Material

Monitoring the Activation of Open Metal Sites in [$Fe_xM_{3-x}(\mu_3$-O)] Cluster-Based Metal–Organic Frameworks by Single Crystal X-Ray Diffraction

Wenmiao Chen,[1,2‡] Zhi Wang,[3‡] Qi Wang,[2] Khaoula El-Yanboui,[4] Kui Tan,[4] Heather M. Barkholtz,[5] Di-Jia Liu,[5] Peiyu Cai,[2] Liang Feng,[2] Youcong Li,[1] Jun-Sheng Qin,[2] Shuai Yuan,[1,2]* Di Sun,[3]* and Hong-Cai Zhou[2,6]*

[1]*State Key Laboratory of Coordination Chemistry, School of Chemistry and Chemical Engineering, Collaborative Innovation Center of Advanced Microstructures, Nanjing University, Nanjing 210093, P. R. China*

[2]*Department of Chemistry, Texas A&M University, College Station, Texas 77843-3255, United States*

[3]*School of Chemistry and Chemical Engineering, Shandong University, Jinan 250100, People's Republic of China*

[4]*Department of Materials Science & Engineering, University of Texas at Dallas, Richardson, Texas 75080, United States*

[5]*Chemical Sciences & Engineering Division, Argonne National Laboratory, Lemont, Illinois, 60439, United States*

[6]*Department of Materials Science and Engineering, Texas A&M University, College Station, Texas 77842, United States*

Correspondence: syuan@nju.edu.cn; dsun@sdu.edu.cn; zhou@chem.tamu.edu

Experimental Details

Materials and Characterization. All the reagents and solvents were commercially available and used as received. The 3,3′,5,5′-azobenzenetetracarboxylic acid (H_4ABTC) was synthesized according to the literature reported procedures.[1] Powder X-ray diffraction (PXRD) was carried out with a Bruker D8-Focus Bragg-Brentano X-ray Powder Diffractometer equipped with a Cu sealed tube (λ = 1.54178 Å) at 40 kV and 40 mA. The magnetic measurements were performed using a Quantum Design MPMS-SQUID-VSM magnetometer. Single crystal X-ray diffraction (SC-XRD) was measured on a Rigaku Oxford Diffraction XtaLAB Synergy-S diffractometer equipped with a Cu-Kα sealed-tube X-ray source (λ = 1.54184 Å). Scanning electron microscopy with energy dispersive X-ray spectroscopy (SEM/EDX) was performed on an FEI Quanta 600 field emission scanning electron microscope (America). N_2 sorption measurements were conducted using a Micrometritics ASAP 2020 system.

Synthesis of PCN-250 (Fe). PCN-250 (Fe) was synthesized following the literature with modification.[2] A mixture of $Fe(NO_3)_3 \cdot 9H_2O$ (500 mg, 1.23 mmol), H_4ABTC (100 mg, 0.28 mmol), acetic acid (10 mL), and DMF (20 mL) was charged in a Pyrex vial and heated in an oven at 150°C for 24 h. After cooling to room temperature, dark red crystals were collected by centrifugation.

Synthesis of PCN-250 (Fe_2Ni). A mixture of $Fe(NO_3)_3 \cdot 9H_2O$ (300 mg, 0.74 mmol), $Ni(NO_3)_2$ $6H_2O$ (150 mg, 0.51 mmol), H_4ABTC (100 mg, 0.28 mmol), acetic acid (10 mL), and DMF (20 mL) was charged in a Pyrex vial and heated in an oven at 150°C for 24 h. After cooling to room temperature, dark red crystals were collected by centrifugation. The Ni/(Ni+Fe) ratio was determined to be 0.303 based on ICP-MS.

Synthesis of PCN-250 ($FeNi_2$). A mixture of $Fe(NO_3)_3 \cdot 9H_2O$ (100 mg, 0.24), $Ni(NO_3)_2 \cdot 6H_2O$ (290 mg, 1.00 mmol), H_4ABTC (100 mg, 0.28 mmol), acetic acid (10 mL), and DMF (20 mL) was charged in a Pyrex vial and heated in an oven at 150°C for 24 h. After cooling to room temperature, dark red crystals were collected by centrifugation. The Ni/(Ni+Fe) ratio was determined to be 0.663 based on ICP-MS.

Synthesis of PCN-250 (Fe_2Co). A mixture of $Fe(NO_3)_3 \cdot 9H_2O$ (100 mg, 0.24), $Co(NO_3)_2 \cdot 6H_2O$ (400 mg, 1.37 mmol), H_4ABTC (100 mg, 0.28 mmol), acetic acid (10 mL), and DMF (20 mL) was charged in a Pyrex vial and heated in an oven at 150°C for 24 h. After cooling to room temperature, dark red crystals were collected by centrifugation. The Co/(Co+Fe) ratio was determined to be 0.286 based on ICP-MS.

Synthesis of PCN-250 (Fe_2Zn). A mixture of $Fe(NO_3)_3 \cdot 9H_2O$ (100 mg, 0.24), $Zn(NO_3)_2 \cdot 6H_2O$ (500 mg, 1.34 mmol), H_4ABTC (100 mg, 0.28 mmol), acetic acid (10 mL), and DMF (20 mL) was charged in a Pyrex vial and heated in an oven at 150°C for 24 h. After cooling to room temperature, dark red crystals were collected by centrifugation. The Zn/(Zn+Fe) ratio was determined to be 0.263 based on ICP-MS.

Synthesis of PCN-250 (Fe_2Mg). A mixture of $Fe(NO_3)_3 \cdot 9H_2O$ (100 mg, 0.24), $Mg(NO_3)_2 \cdot 6H_2O$ (400 mg, 1.56 mmol), H_4ABTC (100 mg, 0.28 mmol), acetic acid (10 mL), and DMF (20 mL) was charged in a Pyrex vial and heated in an oven at 150°C for 24 h. After cooling to room

temperature, dark red crystals were collected by centrifugation. The Mg/(Mg+Fe) ratio was determined to be 0.298 based on ICP-MS.

X-ray absorption spectroscopy (XAS). X-ray absorption measurements (XAS) including X-ray absorption near edge structure (XANES) and extended X-ray absorption fine structure (EXAFS) spectroscopy were performed at the beamline12-BM of the Advanced Photon Source (APS) at Argonne National Laboratory. The XAS spectra were collected at the Fe K-edge (7.1109 KeV) using transmission mode. A Fe foil EXAFS was measured with the aid of a fluorescence detector for energy calibration for each scan of the samples. For this experiment we designed an *in situ* reactor consisted of a quartz tube flow reactor and a stainless steel sample holder. During the experiment, a constant flow of high purity helium was used to mimic thermal activation in vacuum while the temperature of the furnace was raised from room temperature under controlled temperature ramp. XAS spectroscopic data were taken continuously as the temperature increased to record the oxidation state and coordination structural changes.

DFT calculations details: To study the differential adsorption of gaseous N_2 on PCN-250(Fe_3), PCN-250(Fe_2Ni), and PCN-250($FeNi_2$), the cluster models were taken from the single-crystal structures of PCN-250 series containing 6 benzoate ligands and trinuclear metal cluster.

DFT calculations were carried out using the dmol3 module in the Materials Studio software package. The structures were energy minimized using the GGA PBE functional with the Grimme method for DFT-D correction. A proton was attached to the center oxo group to balance the charge in the cluster model for PCN-250($FeNi_2$). A hydroxy group was added to one of the three Fe centers to balance the charge in the cluster model for PCN-250(Fe_3).

The heat of adsorption was defined as E_{ads} = E(cluster-N_2) – E(cluster) – E(N_2), where E(cluster-N_2), E(cluster), and E(N_2) represent the energy for the cluster-N_2 system after adsorption, the cluster without N_2, and nitrogen molecule, respectively. The E(cluster-N_2), E(cluster), E(N_2) were collected by separate energy minimizations. The Heat of adsorption was then calculated by dividing the E_{ads} by the corresponding number of N_2 molecules being adsorbed. 2 in PCN-250(Fe_3), 3 in PCN-250(Fe_2Ni), and 3 in PCN-250($FeNi_2$)).

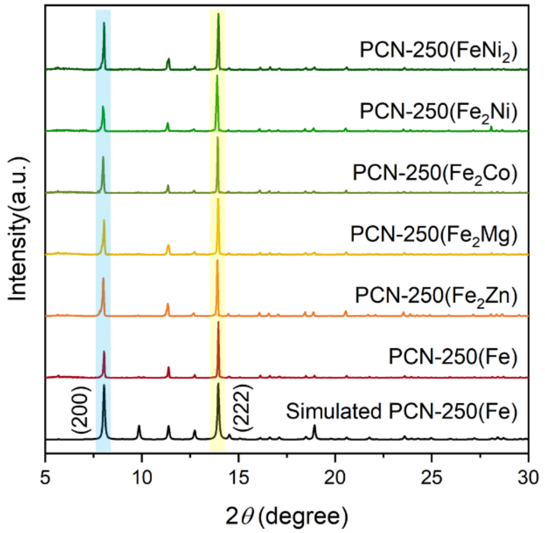

Figure S1. PXRD patterns of as-synthesized PCN-250 (Fe), PCN-250 (Fe_2Mg), PCN-250 (Fe_2Zn), PCN-250 (Fe_2Co), PCN-250 (Fe_2Ni), and PCN-250 ($FeNi_2$). Simulated PXRD was calculated from the crystal structure of PCN-250 (Fe) by applying a preferred orientation along the [111] direction. The higher (222) and lower (200)/(211) diffraction peaks are attributed to the preferred orientation of PCN-250 (FeM) crystals along the [111] direction and unexposed (211) facets.

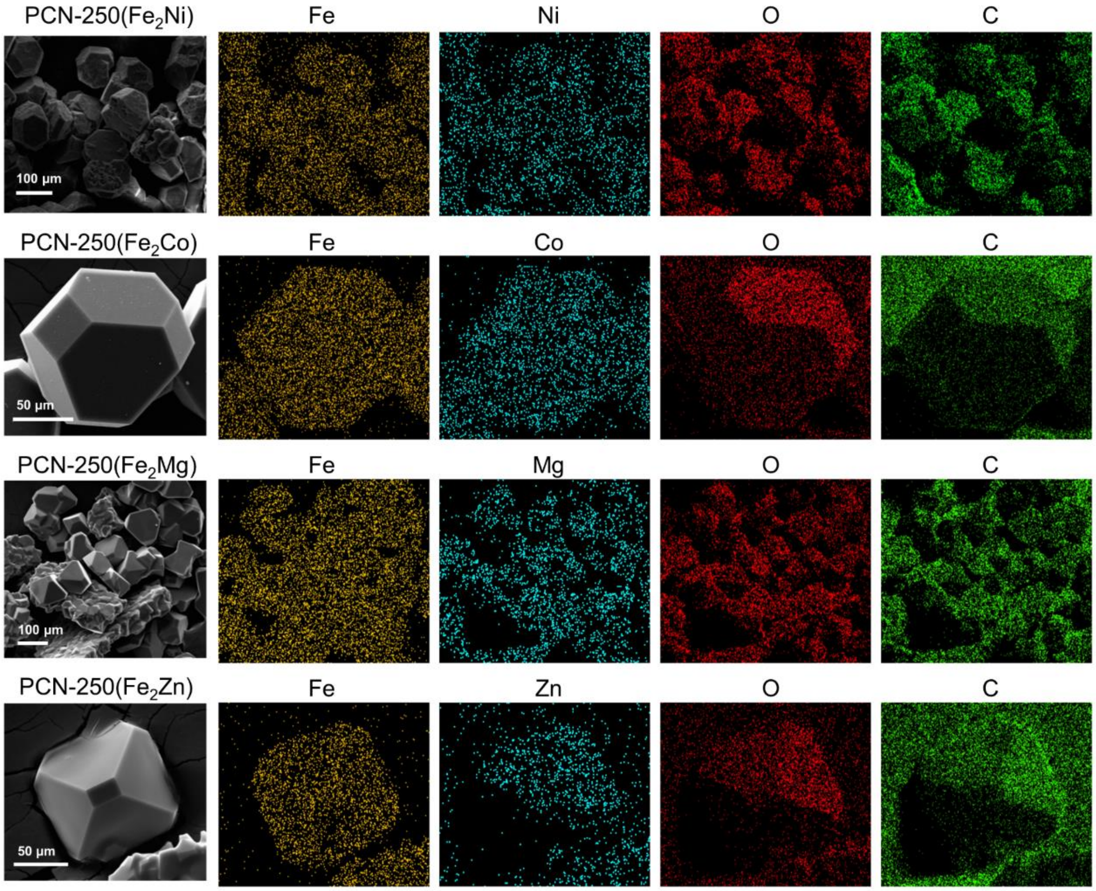

Figure S2. SEM images and element mapping of PCN-250 (Fe_2Mg), PCN-250 (Fe_2Zn), PCN-250 (Fe_2Co), and PCN-250 (Fe_2Ni) showing the uniform distribution of heterometals.

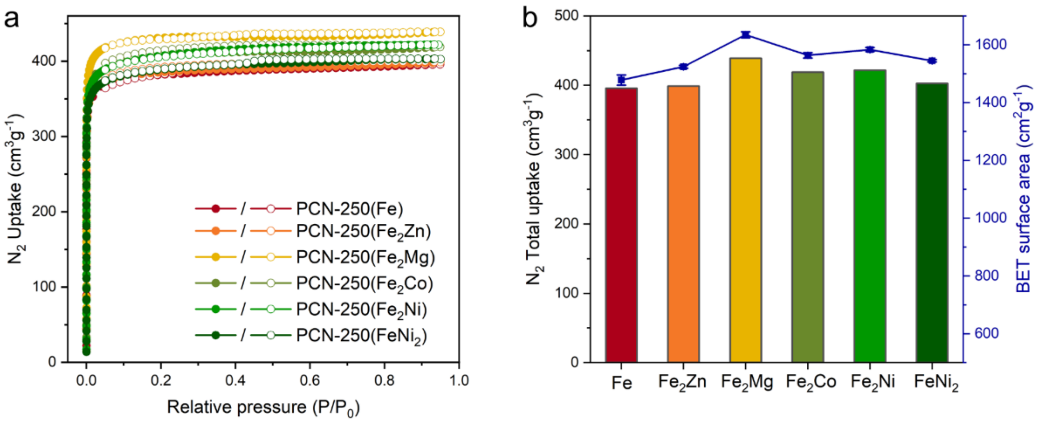

Figure S3. (a) N_2 adsorption-desorption isotherms of PCN-250 (Fe), PCN-250 (Fe_2Mg), PCN-250 (Fe_2Zn), PCN-250 (Fe_2Co), PCN-250 (Fe_2Ni), and PCN-250 ($FeNi_2$) at 77 K. (b) Comparison of N_2 total uptake and BET surface area. Before N_2 adsorption measurements, all the samples were solvent-exchanged by methanol and activated under vacuum at 473 K for 6 h.

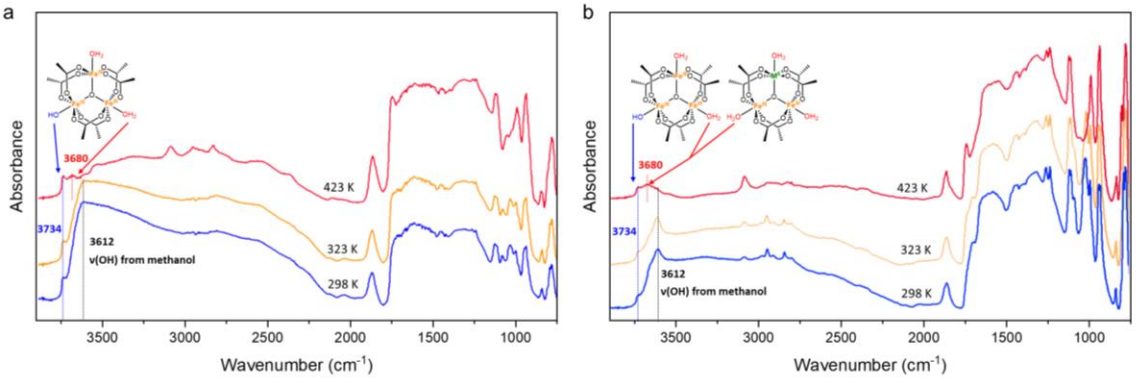

Figure S4. IR spectra of (a) PCN-250 (Fe) and (b) PCN-250 (Fe_2Ni) at 323 K and 423 K showing the existence of terminal –OH/H_2O ligands. PCN-250 (Fe_2Ni) shows a lower ratio of terminal –OH, which is consistent with the structural model. No sign of nitrate was observed at 1380 cm^{-1}, confirming that no nitrate was left in the pore after synthesis.[4]

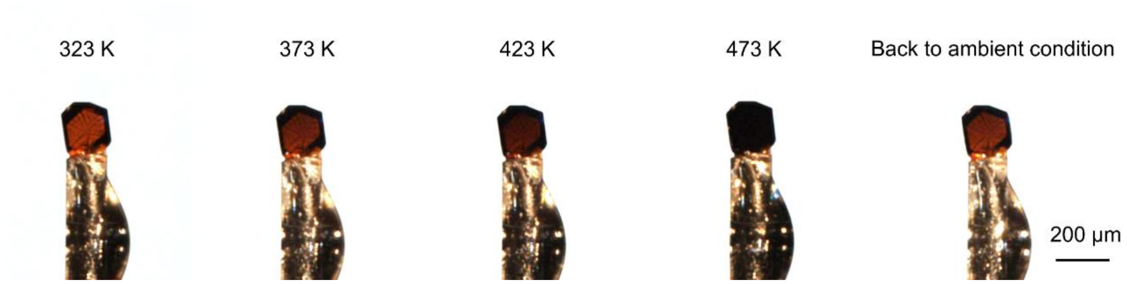

Figure S5. Optical microscopic images of PCN-250 (Fe) crystals at different temperatures. The color change from dark red to black is related to the coordination geometry change of Fe from an octahedron to a square pyramid.

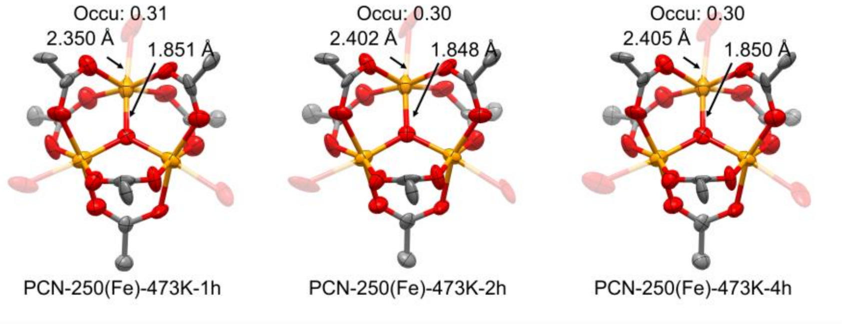

Figure S6. Single crystal structure of the PCN-250 (Fe) crystal heated under 473 K for 1h, 2h and 4h showing the maintained coordination environment of Fe centers (similar bond lengths and terminal O occupancy).

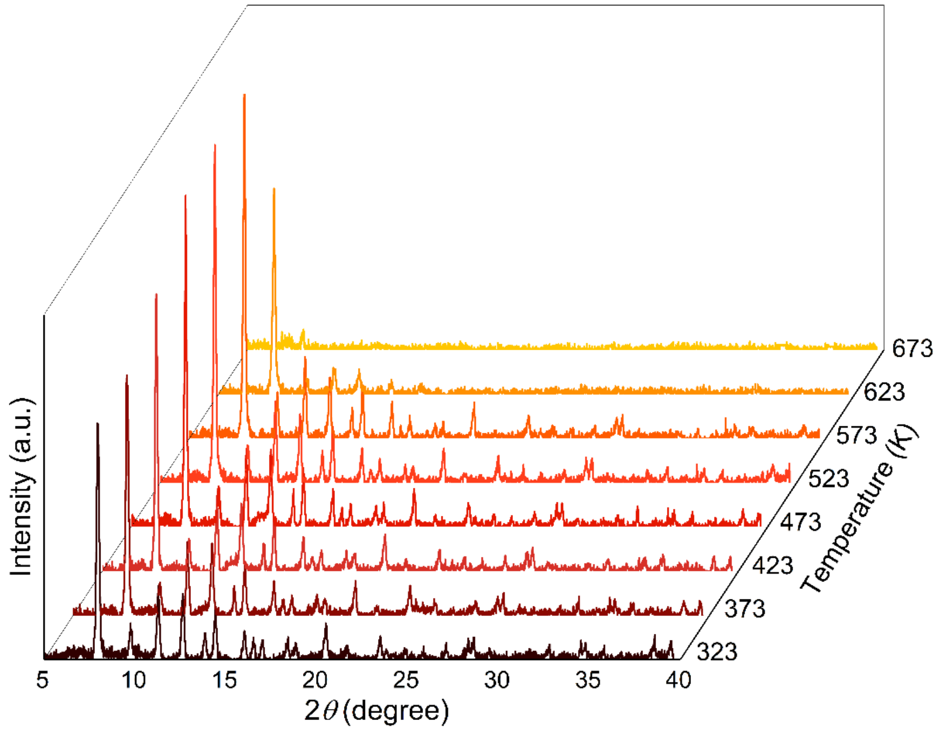

Figure S7. Variable temperature PXRD of PCN-250 (Fe) from 323 K to 673 K in N_2 atmosphere showing the maintained crystallinity up to 573 K.

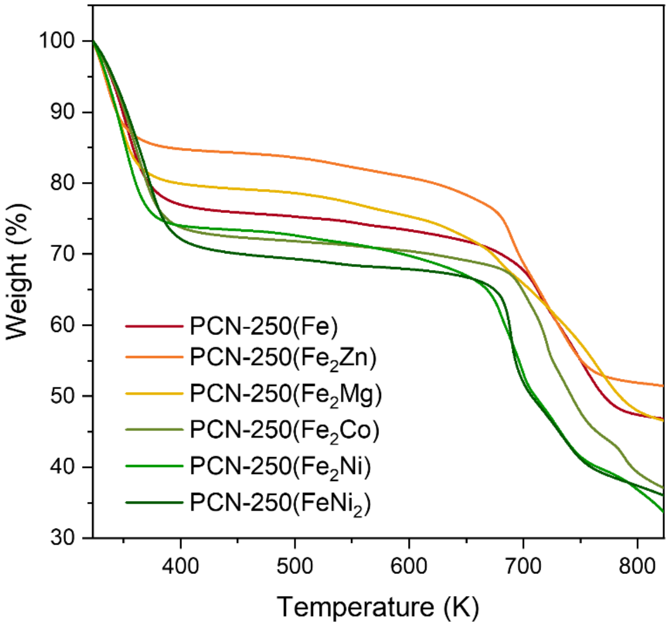

Figure S8. TGA of PCN-250 (Fe), PCN-250 (Fe_2Mg), PCN-250 (Fe_2Zn), PCN-250 (Fe_2Co), PCN-250 (Fe_2Ni), and PCN-250 ($FeNi_2$) in N_2 atmosphere.

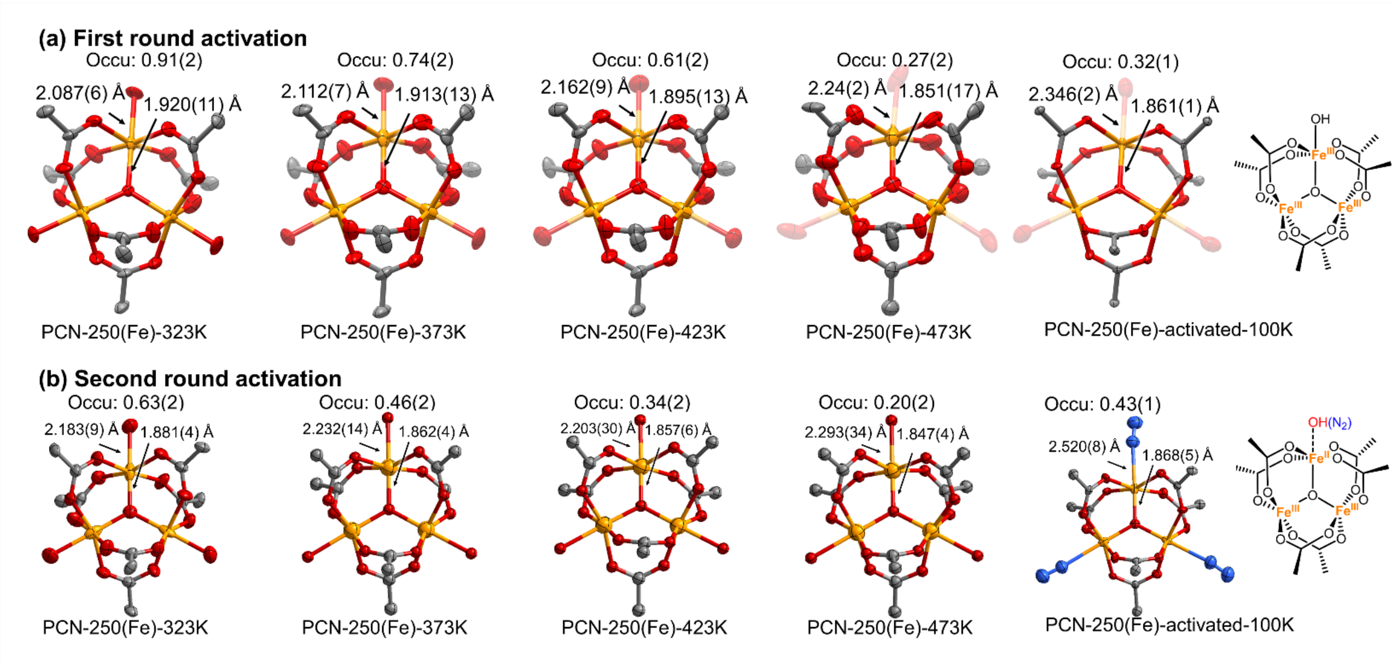

Figure S9. Activation and solvation cycles studied by SCXRD. (a) Single crystal structures of PCN-250(Fe) for the first-round activation at 323 K, 373 K, 423 K, 473 K, and 100 K (after thermal activation). (b) Single-crystal structures of the PCN-250(Fe) for the second-round activation at 323 K, 373 K, 423 K, 473 K, and 100 K (after thermal activation). The activated crystal of PCN-250(Fe) was exposed to air for 12 h before the second-round variable-temperature SCXRD analysis.

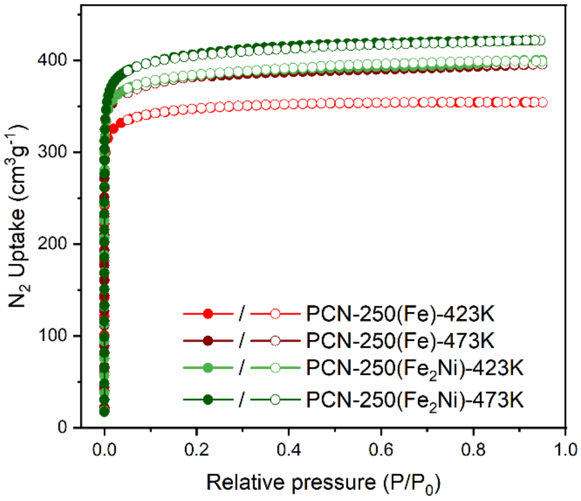

Figure S10. N_2 adsorption-desorption isotherms of PCN-250 (Fe) and PCN-250 (Fe_2Ni) at 77 K. The samples were solvent-exchanged by methanol and activated under vacuum for 6 h at 423 K and 473 K, respectively.

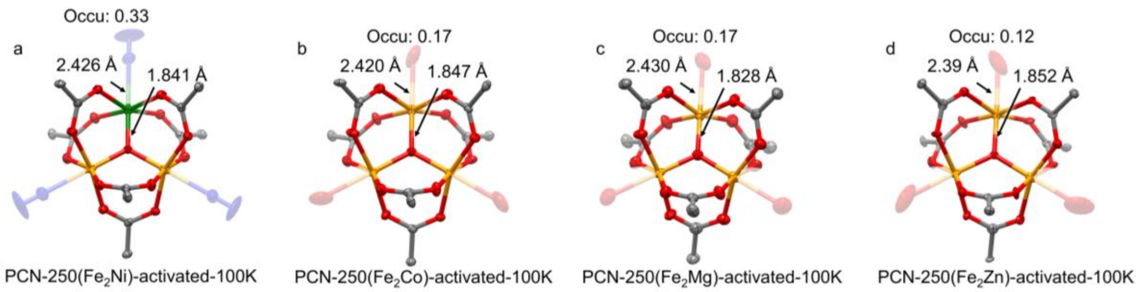

Figure S11. Single crystal structures of activated PCN-250(Fe_2M) (M= Ni^{2+}, Co^{2+}, Zn^{2+}, Mg^{2+}) measured at 100 K. The crystals were activated at 473 K under N_2 flow for 1 h and then cooled to 100 K for SCXRD collection.

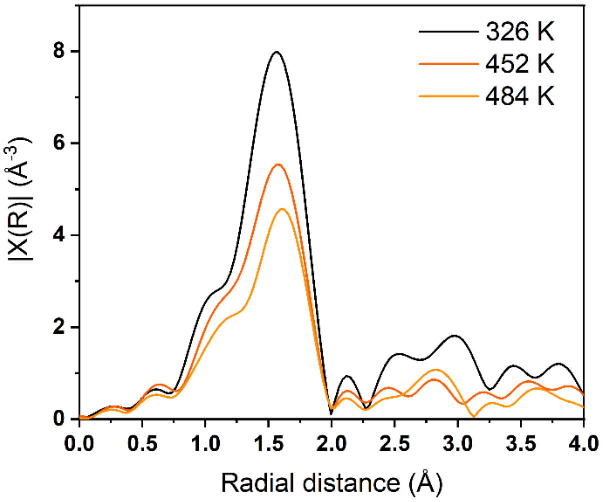

Figure S9. Extended X-ray absorption fine structure (EXAFS) spectra of PCN-250 (Fe) under different temperatures. R is the peak location scale in real space, without phase shift correction.

Table S1. Variable temperature SCXRD data and structure refinements of PCN-250 (Fe).

Name	PCN-250 (Fe) -323K	PCN-250 (Fe) -373K	PCN-250 (Fe) -423K	PCN-250 (Fe) -473K	PCN-250 (Fe) -100K
CCDC	2192488	2192489	2192490	2192491	2192492
Empirical formula	$C_8H_3FeNO_{5.24}$	$C_8H_3FeNO_{5.08}$	$C_8H_3FeNO_{4.94}$	$C_8H_3FeNO_{4.65}$	$C_8H_3FeNO_{4.66}$
Formula weight	252.84	250.25	247.99	243.3	243.47
Temperature/K	322.98(13)	373.01(16)	422.99(19)	473.0(3)	99.97(11)
Crystal system	cubic	cubic	cubic	cubic	cubic
Space group	*P*-43n	*P*-43n	*P*-43n	*P*-43n	*P*-43n
a/Å	21.9076(4)	21.8672(4)	21.8841(4)	21.9012(5)	21.9731(2)
b/Å	21.9076(4)	21.8672(4)	21.8841(4)	21.9012(5)	21.9731(2)
c/Å	21.9076(4)	21.8672(4)	21.8841(4)	21.9012(5)	21.9731(2)
α/°	90	90	90	90	90
β/°	90	90	90	90	90
γ/°	90	90	90	90	90
Volume/Å^3	10514.4(6)	10456.3(6)	10480.6(6)	10505.2(7)	10609.0(3)
Z	24	24	24	24	24
ρ_{calc}g/cm^3	0.958	0.953	0.943	0.923	0.915
μ/mm^{-1}	6.959	6.986	6.96	6.924	6.857
F(000)	3022	2990	2964	2908	2910
Radiation	Cu Kα (λ = 1.54184)	Cu Kα (λ = 1.54184)	Cu Kα (λ = 1.54184)	Cu Kα (λ = 1.54184)	Cu Kα (λ = 1.54184)
2Θ range for data collection/°	8.072 to 134.146	8.086 to 134.052	8.08 to 134.04	9.892 to 134.028	8.048 to 134.126
Index ranges	-10 ≤ h ≤ 14, 0 ≤ k ≤ 18, 2 ≤ l ≤ 26	-14 ≤ h ≤ 9, 0 ≤ k ≤ 18, 2 ≤ l ≤ 26	-14 ≤ h ≤ 9, 0 ≤ k ≤ 18, 2 ≤ l ≤ 26	-12 ≤ h ≤ 14, 0 ≤ k ≤ 18, 2 ≤ l ≤ 26	-10 ≤ h ≤ 14, 0 ≤ k ≤ 18, 2 ≤ l ≤ 26
Reflections collected	2782	2728	2705	2902	2780
Independent reflections	2782 [R_{sigma} = 0.0413]	2728 [R_{sigma} = 0.0462]	2705 [R_{sigma} = 0.0454]	2902 [R_{sigma} = 0.0631]	2780 [R_{sigma} = 0.0390]
Data/restraints/parameters	2782/0/141	2728/0/141	2705/0/141	2902/0/141	2780/0/141
Goodness-of-fit on F^2	1.136	1.113	1.136	1.092	1.138
Final R indexes [I>=2σ (I)]	R_1 = 0.0632, wR_2 = 0.1704	R_1 = 0.0620, wR_2 = 0.1633	R_1 = 0.0560, wR_2 = 0.1540	R_1 = 0.1311, wR_2 = 0.3528	R_1 = 0.0453, wR_2 = 0.1081
Final R indexes [all data]	R_1 = 0.0713, wR_2 = 0.1752	R_1 = 0.0741, wR_2 = 0.1704	R_1 = 0.0688, wR_2 = 0.1604	R_1 = 0.1422, wR_2 = 0.3589	R_1 = 0.0478, wR_2 = 0.1092
Largest diff. peak/hole / e Å^{-3}	1.02/-0.43	0.69/-0.46	0.43/-0.43	0.86/-0.78	0.34/-0.42

$R_1 = \Sigma||F_o| - |F_c||/\Sigma|F_o|$. $wR_2 = |\Sigma w(|F_o|^2 - |F_c|^2)|/\Sigma|w(F_o^2)^2|^{1/2}$.

Table S2. SCXRD data and structure refinements of PCN-250(Fe) heated at 473 K for 2 h and 4 h.

Name	PCN-250 (Fe) -473K-2h	PCN-250 (Fe) -473K-4h
CCDC	2192493	2192494
Empirical formula	$C_8FeNO_{4.64}$	$C_8H_3FeNO_{4.63}$
Formula weight	240.14	243.04
Temperature/K	473.0(3)	473.0(2)
Crystal system	cubic	cubic
Space group	*P*-43n	*P*-43n
a/Å	21.9107(5)	21.9214(4)
b/Å	21.9107(5)	21.9214(4)
c/Å	21.9107(5)	21.9214(4)
α/°	90	90
β/°	90	90
γ/°	90	90
Volume/$Å^3$	10518.9(7)	10534.3(6)
Z	24	24
ρ_{calc}g/cm^3	0.91	0.919
μ/mm^{-1}	6.914	6.904
F(000)	2834	2905
Radiation	Cu Kα (λ = 1.54184)	Cu Kα (λ = 1.54184)
2Θ range for data collection/°	9.888 to 134.108	9.884 to 133.976
Index ranges	-14 ≤ h ≤ 12, 0 ≤ k ≤ 18, 2 ≤ l ≤ 26	-12 ≤ h ≤ 14, 0 ≤ k ≤ 18, 2 ≤ l ≤ 26
Reflections collected	2906	2909
Independent reflections	2906 [R_{sigma} = 0.0625]	2909 [R_{sigma} = 0.0595]
Data/restraints/parameters	2906/0/141	2909/0/141
Goodness-of-fit on F^2	1.115	1.091
Final R indexes [I>=2σ (I)]	R_1 = 0.1389, wR_2 = 0.3706	R_1 = 0.1366, wR_2 = 0.3653
Final R indexes [all data]	R_1 = 0.1476, wR_2 = 0.3759	R_1 = 0.1452, wR_2 = 0.3703
Largest diff. peak/hole / e Å^{-3}	0.89/-0.90	0.90/-0.93

$R_1 = \Sigma||F_o| - |F_c||/\Sigma|F_o|$. $wR_2 = |\Sigma w(|F_o|^2 - |F_c|^2)|/\Sigma|w(F_o^2)^2|^{1/2}$.

Table S3. Variable temperature SCXRD data and structure refinements of PCN-250 (Fe) towards solvation and reactivation.

Name	PCN-250 (Fe) -323K	PCN-250 (Fe) -373K	PCN-250 (Fe) -423K	PCN-250 (Fe) -473K	PCN-250 (Fe) -100K
CCDC	2225979	2225980	2225981	2225982	2225978
Empirical formula	$C_8H_3FeNO_{4.96}$	$C_8H_3FeNO_{4.80}$	$C_8H_3FeNO_{4.68}$	$C_8H_3FeNO_{4.55}$	$C_8H_3FeNO_{4.33}$
Formula weight	248.38	245.70	243.84	241.74	258.67
Temperature/K	322.99(10)	372.99(11)	422.99(10)	472.99(13)	100.01(13)
Crystal system	cubic	Cubic	cubic	cubic	cubic
Space group	*P*-43n	*P*-43n	*P*-43n	*P*-43n	*P*-43n
a/Å	21.8859(3)	21.8827(3)	21.9068(3)	21.9134(2)	22.0083(3)
b/Å	21.8859(3)	21.8827(3)	21.9068(3)	21.9134(2)	22.0083(3))
c/Å	21.8859(3)	21.8827(3)	21.9068(3)	21.9134(2)	22.0083(3)
α/°	90	90	90	90	90
β/°	90	90	90	90	90
γ/°	90	90	90	90	90
Volume/Å^3	10483.2(4)	10478.5(4)	10513.2(4)	10522.8(3)	10660.1(4)
Z	24	24	24	24	24
ρ_{calc}g/cm^3	0.944	0.934	0.924	0.916	0.967
μ/mm^{-1}	6.960	6.952	6.921	6.905	6.858
F(000)	2969	2937	2914	2889	3092
Radiation	Cu Kα (λ = 1.54184)	Cu Kα (λ = 1.54184)	Cu Kα (λ = 1.54184)	Cu Kα (λ = 1.54184)	Cu Kα (λ = 1.54184)
2Θ range for data collection/°	8.072 to 134.018	9.016 to 138.748	9.010 to 139.15	9.892 to 134.028	8.048 to 134.126
Index ranges	-10 ≤ h ≤ 14, 0 ≤ k ≤ 18, 2 ≤ l ≤ 26	-14 ≤ h ≤ 9, 0 ≤ k ≤ 18, 2 ≤ l ≤ 26	-14 ≤ h ≤ 9, 0 ≤ k ≤ 18, 2 ≤ l ≤ 26	-12 ≤ h ≤ 14, 0 ≤ k ≤ 18, 2 ≤ l ≤ 26	-10 ≤ h ≤ 14, 0 ≤ k ≤ 18, 2 ≤ l ≤ 26
Reflections collected	2810	2832	2603	2939	2780
Independent reflections	2810 [R_{sigma} = 0.055]	2728 [R_{sigma} = 0.055]	2603 [R_{sigma} = 0.069]	2939 [R_{sigma} = 0.055]	2780 [R_{sigma} = 0.084]
Data/restraints/parameters	2810/0/141	2728/0/141	2603/0/141	2939/0/141	2780/0/141
Goodness-of-fit on F^2	1.049	1.035	1.100	1.054	1.138
Final R indexes [I>=2σ (I)]	R_1 = 0.0510, wR_2 = 0.1104	R_1 = 0.0526, wR_2 = 0.1233	R_1 = 0.0754, wR_2 = 0.1540	R_1 = 0.0467, wR_2 = 0.1028	R_1 = 0.0673, wR_2 = 0.1081
Final R indexes [all data]	R_1 = 0.0566, wR_2 = 0.1268	R_1 = 0.0595, wR_2 = 0.1356	R_1 = 0.0907, wR_2 = 0.1717	R_1 = 0.0506, wR_2 = 0.1153	R_1 = 0.0688, wR_2 = 0.1715
Largest diff. peak/hole / e Å^{-3}	0.277/-0.381	0.275/-0.493	0.377/-0.435	0.86/-0.78	0.34/-0.42

$R_1 = \Sigma||F_o| - |F_c||/\Sigma|F_o|$. $wR_2 = |\Sigma w(|F_o|^2 - |F_c|^2)|/\Sigma|w(F_o^2)^2|^{1/2}$.

Table S4. Variable temperature SCXRD data and structure refinements of PCN-250 (Fe_2Ni).

Name	PCN-250(Fe_2Ni) -323K	PCN-250(Fe_2Ni) -373K	PCN-250(Fe_2Ni) -423K	PCN-250(Fe_2Ni) -473K	PCN-250(Fe_2Ni) -100K
CCDC	2192496	2192497	2192498	2192499	2192500
Empirical formula	$C_8H_3Fe_{0.67}NNi_{0.33}O_{5.19}$	$C_8H_3Fe_{0.67}NNi_{0.33}O_{5.03}$	$C_8H_3Fe_{0.67}NNi_{0.33}O_{4.74}$	$C_8H_3Fe_{0.67}NNi_{0.33}O_{4.43}$	$C_8H_3Fe_{0.67}N_{1.56}Ni_{0.33}O_{4.33}$
Formula weight	252.93	250.38	245.68	240.78	247.14
Temperature/K	322.98(11)	373.00(16)	423.01(18)	472.9(2)	99.97(11)
Crystal system	cubic	cubic	cubic	cubic	cubic
Space group	*P*-43n	*P*-43n	*P*-43n	*P*-43n	*P*-43n
a/Å	21.9579(2)	21.9246(2)	21.8929(2)	21.8734(2)	21.9237(2)
b/Å	21.9579(2)	21.9246(2)	21.8929(2)	21.8734(2)	21.9237(2)
c/Å	21.9579(2)	21.9246(2)	21.8929(2)	21.8734(2)	21.9237(2)
α/°	90	90	90	90	90
β/°	90	90	90	90	90
γ/°	90	90	90	90	90
Volume/$Å^3$	10587.0(3)	10538.9(3)	10493.2(3)	10465.2(3)	10537.6(3)
Z	24	24	24	24	24
$\rho_{calc}g/cm^3$	0.952	0.947	0.933	0.917	0.935
μ/mm^{-1}	5.152	5.164	5.166	5.159	5.139
F(000)	3028	2998	2941	2882	2959
Radiation	Cu Kα (λ = 1.54184)	Cu Kα (λ = 1.54184)	Cu Kα (λ = 1.54184)	Cu Kα (λ = 1.54184)	Cu Kα (λ = 1.54184)
2Θ range for data collection/°	9.866 to 134.116	9.02 to 134.134	9.032 to 134.13	9.04 to 133.974	9.02 to 134.144
Index ranges	-16 ≤ h ≤ 20, -25 ≤ k ≤ 18, -6 ≤ l ≤ 26	-16 ≤ h ≤ 20, -18 ≤ k ≤ 25, -26 ≤ l ≤ 6	-16 ≤ h ≤ 20, -25 ≤ k ≤ 18, -6 ≤ l ≤ 26	-16 ≤ h ≤ 20, -25 ≤ k ≤ 18, -6 ≤ l ≤ 26	-20 ≤ h ≤ 16, -18 ≤ k ≤ 25, -6 ≤ l ≤ 26
Reflections collected	10979	11053	10895	10852	10919
Independent reflections	2986 [R_{int} = 0.0391, R_{sigma} = 0.0425]	2994 [R_{int} = 0.0427, R_{sigma} = 0.0464]	3005 [R_{int} = 0.0406, R_{sigma} = 0.0349]	2992 [R_{int} = 0.0399, R_{sigma} = 0.0345]	2968 [R_{int} = 0.0429, R_{sigma} = 0.0438]
Data/restraints/parameters	2986/0/140	2994/0/140	3005/0/140	2992/0/140	2968/0/150
Goodness-of-fit on F^2	1.03	1.041	1.024	1.039	1.012
Final R indexes [I>=2σ (I)]	R_1 = 0.0382, wR_2 = 0.1032	R_1 = 0.0347, wR_2 = 0.0973	R_1 = 0.0351, wR_2 = 0.0865	R_1 = 0.0307, wR_2 = 0.0692	R_1 = 0.0296, wR_2 = 0.0642
Final R indexes [all data]	R_1 = 0.0439, wR_2 = 0.1058	R_1 = 0.0416, wR_2 = 0.0999	R_1 = 0.0413, wR_2 = 0.0897	R_1 = 0.0369, wR_2 = 0.0718	R_1 = 0.0344, wR_2 = 0.0655
Largest diff. peak/hole / e $Å^{-3}$	0.61/-0.28	0.47/-0.25	0.26/-0.36	0.17/-0.21	0.23/-0.29

$R_1 = \Sigma||F_o| - |F_c||/\Sigma|F_o|$. $wR_2 = |\Sigma w(|F_o|^2 - |F_c|^2)|/\Sigma|w(F_o^2)^2|^{1/2}$.

Table S5. Variable temperature SCXRD data and structure refinements of PCN-250 ($FeNi_2$).

Name	PCN-250($FeNi_2$) -323K	PCN-250($FeNi_2$) -373K	PCN-250($FeNi_2$) -423K	PCN-250($FeNi_2$) -473K	PCN-250($FeNi_2$) -100K
CCDC	2192501	2192502	2192503	2192504	2192505
Empirical formula	$C_8H_3Fe_{0.33}NNi_{0.67}O_{5.15}$	$C_8H_3Fe_{0.33}NNi_{0.67}O_{4.83}$	$C_8H_3Fe_{0.33}NNi_{0.67}O_{4.55}$	$C_8H_3Fe_{0.33}NNi_{0.67}O_{4.33}$	$C_8H_3Fe_{0.33}N_{2.78}Ni_{0.67}O_{4.33}$
Formula weight	254.06	248.07	243.68	238.19	265.1
Temperature/K	322.99(13)	372.99(17)	422.99(19)	473.0(3)	100.03(10)
Crystal system	cubic	cubic	cubic	cubic	cubic
Space group	*P*-43n	*P*-43n	*P*-43n	*P*-43n	*P*-43n
a/Å	21.97510(16)	21.9224(2)	21.88360(10)	21.8548(2)	21.9283(4)
b/Å	21.97510(16)	21.9224(2)	21.88360(10)	21.8548(2)	21.9283(4)
c/Å	21.97510(16)	21.9224(2)	21.88360(10)	21.8548(2)	21.9283(4)
α/°	90	90	90	90	90
β/°	90	90	90	90	90
γ/°	90	90	90	90	90
Volume/$Å^3$	10611.9(2)	10535.8(3)	10479.88(14)	10438.6(3)	10544.2(6)
Z	24	24	24	24	24
$\rho_{calc}g/cm^3$	0.954	0.938	0.927	0.909	1.002
μ/mm^{-1}	3.387	3.387	3.386	3.384	3.42
F(000)	3046	2974	2922	2832	3179
Radiation	Cu Kα (λ = 1.54184)	Cu Kα (λ = 1.54184)	Cu Kα (λ = 1.54184)	Cu Kα (λ = 1.54184)	Cu Kα (λ = 1.54184)
2Θ range for data collection/°	8.046 to 134.062	8.066 to 134.16	8.08 to 134.046	8.092 to 134.006	8.064 to 134.088
Index ranges	-20 ≤ h ≤ 26, -24 ≤ k ≤ 17, -5 ≤ l ≤ 22	-20 ≤ h ≤ 26, -24 ≤ k ≤ 17, -4 ≤ l ≤ 22	-17 ≤ h ≤ 23, -26 ≤ k ≤ 20, -22 ≤ l ≤ 4	-23 ≤ h ≤ 17, -20 ≤ k ≤ 26, -22 ≤ l ≤ 4	-22 ≤ h ≤ 4, -26 ≤ k ≤ 20, -24 ≤ l ≤ 17
Reflections collected	10342	10288	10177	10011	10039
Independent reflections	3074 [R_{int} = 0.0316, R_{sigma} = 0.0316]	3051 [R_{int} = 0.0336, R_{sigma} = 0.0327]	3024 [R_{int} = 0.0311, R_{sigma} = 0.0310]	2980 [R_{int} = 0.1044, R_{sigma} = 0.0802]	3018 [R_{int} = 0.0903, R_{sigma} = 0.0718]
Data/restraints/parameters	3074/0/141	3051/0/140	3024/0/140	2980/0/131	3018/1/150
Goodness-of-fit on F^2	1.089	1.045	1.06	1.04	1.052
Final R indexes [I>=2σ (I)]	R_1 = 0.0405, wR_2 = 0.1195	R_1 = 0.0375, wR_2 = 0.0987	R_1 = 0.0331, wR_2 = 0.0828	R_1 = 0.0566, wR_2 = 0.1372	R_1 = 0.0650, wR_2 = 0.1693
Final R indexes [all data]	R_1 = 0.0434, wR_2 = 0.1218	R_1 = 0.0407, wR_2 = 0.1006	R_1 = 0.0368, wR_2 = 0.0854	R_1 = 0.0642, wR_2 = 0.1423	R_1 = 0.0730, wR_2 = 0.1786
Largest diff. peak/hole / e $Å^{-3}$	0.69/-0.21	0.45/-0.30	0.21/-0.22	0.60/-0.38	1.60/-0.45

$R_1 = \Sigma||F_o| - |F_c||/\Sigma|F_o|$. $wR_2 = |\Sigma w(|F_o|^2 - |F_c|^2)|/\Sigma|w(F_o^2)^2|^{1/2}$.

Table S6. Calculated energy of PCN-250-N_2 model and heat of adsorption.

N_2 energy<br>-109.4493312		Heat of Adsorption (Hatree)	Heat of Adsorption (kJ/mol)
Fe_3<br>-3042.741087	Fe_3 $2N_2$<br>-3261.66519	-0.01272025	-33
Fe_2Ni<br>-3013.971539	Fe_2Ni $3N_2$<br>-3342.366337	-0.015601433	-41
$FeNi_2$<br>-3061.593168	$FeNi_2$ $3N_2$<br>-3389.989598	-0.0161455	-42

Table S7. Variable temperature SCXRD data and structure refinements of PCN-250 (Fe_2Mg).

Name	PCN-250(Fe_2Mg) -323K	PCN-250(Fe_2Mg) -373K	PCN-250(Fe_2Mg) -423K	PCN-250(Fe_2Mg) -473K	PCN-250(Fe_2Mg) -100K
CCDC	2192506	2192507	2192508	2192509	2192510
Empirical formula	$C_8H_3Fe_{0.67}Mg_{0.33}NO_{5.11}$	$C_8H_3Fe_{0.67}Mg_{0.33}NO_{4.87}$	$C_8H_3Fe_{0.67}Mg_{0.33}NO_{4.67}$	$C_8H_3Fe_{0.67}Mg_{0.33}NO_{4.48}$	$C_8H_3Fe_{0.67}Mg_{0.33}NO_{4.5}$
Formula weight	240.18	236.44	233.15	230.18	230.51
Temperature/K	322.97(13)	372.99(18)	423.0(2)	472.9(2)	100.01(16)
Crystal system	cubic	cubic	cubic	cubic	cubic
Space group	*P*-43n	*P*-43n	*P*-43n	*P*-43n	*P*-43n
a/Å	21.9525(4)	21.9252(4)	21.8945(4)	21.8678(5)	21.8928(5)
b/Å	21.9525(4)	21.9252(4)	21.8945(4)	21.8678(5)	21.8928(5)
c/Å	21.9525(4)	21.9252(4)	21.8945(4)	21.8678(5)	21.8928(5)
α/°	90	90	90	90	90
β/°	90	90	90	90	90
γ/°	90	90	90	90	90
Volume/$Å^3$	10579.2(6)	10539.8(6)	10495.5(6)	10457.2(7)	10493.1(7)
Z	24	24	24	24	24
ρ_{calc}g/cm^3	0.905	0.894	0.885	0.877	0.875
μ/mm^{-1}	4.912	4.914	4.92	4.925	4.91
F(000)	2885	2840	2800	2765	2769
Radiation	Cu Kα (λ = 1.54184)	Cu Kα (λ = 1.54184)	Cu Kα (λ = 1.54184)	Cu Kα (λ = 1.54184)	Cu Kα (λ = 1.54184)
2Θ range for data collection/°	8.056 to 133.79	8.066 to 134.126	8.076 to 134.11	8.086 to 134.044	8.078 to 134.13
Index ranges	-26 ≤ h ≤ 11, -24 ≤ k ≤ 21, -19 ≤ l ≤ 22	-11 ≤ h ≤ 26, -22 ≤ k ≤ 19, -21 ≤ l ≤ 24	-11 ≤ h ≤ 26, -19 ≤ k ≤ 22, -24 ≤ l ≤ 20	-11 ≤ h ≤ 26, -19 ≤ k ≤ 22, -24 ≤ l ≤ 19	-20 ≤ h ≤ 24, -26 ≤ k ≤ 11, -19 ≤ l ≤ 22
Reflections collected	10657	10655	10549	10448	10511
Independent reflections	2923 [R_{int} = 0.0723, R_{sigma} = 0.0697]	2927 [R_{int} = 0.0757, R_{sigma} = 0.0738]	2914 [R_{int} = 0.0777, R_{sigma} = 0.0749]	2901 [R_{int} = 0.0863, R_{sigma} = 0.0717]	2909 [R_{int} = 0.0798, R_{sigma} = 0.0729]
Data/restraints/parameters	2923/0/140	2927/0/140	2914/0/140	2901/0/140	2909/0/140
Goodness-of-fit on F^2	1.101	1.094	1.063	1.053	1.054
Final R indexes [I>=2σ (I)]	R_1 = 0.0583, wR_2 = 0.1410	R_1 = 0.0550, wR_2 = 0.1243	R_1 = 0.0536, wR_2 = 0.1170	R_1 = 0.0528, wR_2 = 0.1130	R_1 = 0.0495, wR_2 = 0.1037
Final R indexes [all data]	R_1 = 0.0773, wR_2 = 0.1662	R_1 = 0.0751, wR_2 = 0.1448	R_1 = 0.0783, wR_2 = 0.1356	R_1 = 0.0869, wR_2 = 0.1405	R_1 = 0.0695, wR_2 = 0.1226
Largest diff. peak/hole / e Å^{-3}	0.57/-0.56	0.36/-0.54	0.30/-0.48	0.18/-0.40	0.25/-0.42

$R_1 = \Sigma||F_o| - |F_c||/\Sigma|F_o|$. $wR_2 = |\Sigma w(|F_o|^2 - |F_c|^2)|/\Sigma|w(F_o^2)^2|^{1/2}$.

Table S8. Variable temperature SCXRD data and structure refinements of PCN-250 (Fe_2Zn).

Name	PCN-250(Fe_2Zn) -323K	PCN-250(Fe_2Zn) -373K	PCN-250(Fe_2Zn) -423K	PCN-250(Fe_2Zn) -473K	PCN-250(Fe_2Zn) -100K
CCDC	2192511	2192512	2192513	2192514	2192515
Empirical formula	$C_8H_3Fe_{0.67}NO_{5.29}Zn_{0.33}$	$C_8H_3Fe_{0.67}NO_{5.2}Zn_{0.33}$	$C_8H_3Fe_{0.67}NO_{4.84}Zn_{0.33}$	$C_8H_3Fe_{0.67}NO_{4.46}Zn_{0.33}$	$C_8H_3Fe_{0.67}NO_{4.52}Zn_{0.33}$
Formula weight	256.82	255.26	249.53	243.47	244.49
Temperature/K	322.97(16)	372.98(19)	423.0(2)	473.0(3)	100.01(10)
Crystal system	cubic	cubic	cubic	cubic	cubic
Space group	*P*-43n	*P*-43n	*P*-43n	*P*-43n	*P*-43n
a/Å	21.9518(3)	21.9434(4)	21.8955(5)	21.9104(4)	21.9678(4)
b/Å	21.9518(3)	21.9434(4)	21.8955(5)	21.9104(4)	21.9678(4)
c/Å	21.9518(3)	21.9434(4)	21.8955(5)	21.9104(4)	21.9678(4)
α/°	90	90	90	90	90
β/°	90	90	90	90	90
γ/°	90	90	90	90	90
Volume/Å^3	10578.2(4)	10566.0(6)	10497.0(7)	10518.4(6)	10601.3(6)
Z	24	24	24	24	24
ρ_{calc}g/cm^3	0.968	0.963	0.947	0.922	0.919
μ/mm^{-1}	5.279	5.278	5.288	5.251	5.214
F(000)	3064	3045	2977	2904	2916
Radiation	Cu Kα (λ = 1.54184)	Cu Kα (λ = 1.54184)	Cu Kα (λ = 1.54184)	Cu Kα (λ = 1.54184)	Cu Kα (λ = 1.54184)
2Θ range for data collection/°	9.008 to 133.798	9.012 to 133.902	9.032 to 134.098	9.024 to 134.11	9.002 to 133.994
Index ranges	-10 ≤ h ≤ 26, -25 ≤ k ≤ 19, -20 ≤ l ≤ 17	-10 ≤ h ≤ 26, -17 ≤ k ≤ 20, -25 ≤ l ≤ 19	-10 ≤ h ≤ 26, -24 ≤ k ≤ 19, -20 ≤ l ≤ 17	-10 ≤ h ≤ 26, -24 ≤ k ≤ 19, -20 ≤ l ≤ 17	-10 ≤ h ≤ 26, -17 ≤ k ≤ 20, -25 ≤ l ≤ 19
Reflections collected	10772	10833	10748	10715	10787
Independent reflections	3042 [R_{int} = 0.0581, R_{sigma} = 0.0533]	3019 [R_{int} = 0.0640, R_{sigma} = 0.0738]	3002 [R_{int} = 0.0723, R_{sigma} = 0.0817]	3021 [R_{int} = 0.0573, R_{sigma} = 0.0516]	2989 [R_{int} = 0.0658, R_{sigma} = 0.0734]
Data/restraints/parameters	3042/0/140	3019/0/140	3002/0/140	3021/0/140	2989/0/140
Goodness-of-fit on F^2	1.094	1.105	1.105	1.053	1.121
Final R indexes [I>=2σ (I)]	R_1 = 0.0603, wR_2 = 0.1659	R_1 = 0.0535, wR_2 = 0.1412	R_1 = 0.0490, wR_2 = 0.1163	R_1 = 0.0438, wR_2 = 0.0909	R_1 = 0.0443, wR_2 = 0.0943
Final R indexes [all data]	R_1 = 0.0796, wR_2 = 0.2090	R_1 = 0.0720, wR_2 = 0.1677	R_1 = 0.0694, wR_2 = 0.1423	R_1 = 0.0642, wR_2 = 0.1182	R_1 = 0.0603, wR_2 = 0.1193
Largest diff. peak/hole / e Å^{-3}	1.04/-0.53	0.77/-0.36	0.39/-0.52	0.26/-0.32	0.35/-0.52

$R_1 = \Sigma||F_o| - |F_c||/\Sigma|F_o|$. $wR_2 = |\Sigma w(|F_o|^2 - |F_c|^2)|/\Sigma|w(F_o^2)^2|^{1/2}$.

Table S9. Variable temperature SCXRD data and structure refinements of PCN-250 (Fe_2Co).

Name	PCN-250(Fe_2Co)-323K	PCN-250(Fe_2Co)-373K	PCN-250(Fe_2Co)-423K	PCN-250(Fe_2Co)-473K	PCN-250(Fe_2Co)-100K
CCDC	2192517	2192518	2192519	2192520	2192521
Empirical formula	$C_8H_3Co_{0.33}Fe_{0.67}NO_{5.21}$	$C_8H_3Co_{0.33}Fe_{0.67}NO_{4.81}$	$C_8H_3Co_{0.33}Fe_{0.67}NO_{4.57}$	$C_8H_3Co_{0.33}Fe_{0.67}NO_{4.49}$	$C_8H_3Co_{0.33}Fe_{0.67}NO_{4.5}$
Formula weight	253.3	246.94	243.14	241.88	241.97
Temperature/K	322.98(14)	372.98(18)	423.0(3)	473.0(4)	100.00(10)
Crystal system	cubic	cubic	cubic	cubic	cubic
Space group	*P*-43n	*P*-43n	*P*-43n	*P*-43n	*P*-43n
a/Å	21.9907(3)	21.9459(2)	21.9164(3)	21.8980(3)	21.9156(5)
b/Å	21.9907(3)	21.9459(2)	21.9164(3)	21.8980(3)	21.9156(5)
c/Å	21.9907(3)	21.9459(2)	21.9164(3)	21.8980(3)	21.9156(5)
α/°	90	90	90	90	90
β/°	90	90	90	90	90
γ/°	90	90	90	90	90
Volume/$Å^3$	10634.5(4)	10569.6(3)	10527.1(4)	10500.6(4)	10525.9(7)
Z	24	24	24	24	24
$\rho_{calc}g/cm^3$	0.949	0.931	0.92	0.918	0.916
μ/mm^{-1}	7.134	7.15	7.163	7.175	7.158
F(000)	3024	2947	2902	2887	2888
Radiation	Cu Kα (λ = 1.54184)	Cu Kα (λ = 1.54184)	Cu Kα (λ = 1.54184)	Cu Kα (λ = 1.54184)	Cu Kα (λ = 1.54184)
2Θ range for data collection/°	8.992 to 134.104	9.01 to 133.87	9.022 to 134.036	9.03 to 134.066	9.022 to 134.046
Index ranges	$-25 \le h \le 20$, $-26 \le k \le 14$, $-8 \le l \le 25$	$-14 \le h \le 26$, $-20 \le k \le 25$, $-25 \le l \le 8$	$-25 \le h \le 20$, $-14 \le k \le 26$, $-25 \le l \le 8$	$-14 \le h \le 26$, $-25 \le k \le 8$, $-25 \le l \le 20$	$-12 \le h \le 26$, $-25 \le k \le 8$, $-25 \le l \le 20$
Reflections collected	10680	10613	10356	10477	10514
Independent reflections	2998 [R_{int} = 0.0760, R_{sigma} = 0.0641]	2969 [R_{int} = 0.0837, R_{sigma} = 0.0722]	2938 [R_{int} = 0.1361, R_{sigma} = 0.1067]	2942 [R_{int} = 0.1004, R_{sigma} = 0.0837]	2907 [R_{int} = 0.0987, R_{sigma} = 0.0749]
Data/restraints/parameters	2998/0/140	2969/0/140	2938/0/140	2942/0/140	2907/0/140
Goodness-of-fit on F^2	1.035	1.043	1.066	1.06	1.066
Final R indexes [I>=2σ (I)]	R_1 = 0.0599, wR_2 = 0.1596	R_1 = 0.0739, wR_2 = 0.1891	R_1 = 0.0706, wR_2 = 0.1715	R_1 = 0.0633, wR_2 = 0.1537	R_1 = 0.0529, wR_2 = 0.1328
Final R indexes [all data]	R_1 = 0.0730, wR_2 = 0.1771	R_1 = 0.0894, wR_2 = 0.2155	R_1 = 0.0861, wR_2 = 0.1926	R_1 = 0.0852, wR_2 = 0.1807	R_1 = 0.0671, wR_2 = 0.1503
Largest diff. peak/hole / e $Å^{-3}$	0.67/-0.44	0.75/-0.55	0.44/-0.61	0.64/-0.44	0.61/-0.48

$R_1 = \Sigma||F_o| - |F_c||/\Sigma|F_o|$. $wR_2 = |\Sigma w(|F_o|^2 - |F_c|^2)|/\Sigma|w(F_o^2)^2|^{1/2}$.

Table S10. EXAFS data analysis of PCN-250(Fe) measured at different temperatures.

**Temperature (K)**	**Path**	**CN**	**R (Å)**	**$\Delta\sigma^2$**	**Δe^o (eV)**	**Temperature (ºC)**	**Overall CN**	**Average R**
**326**	Fe-O	1	1.902	0.00681	0.135	53	6	1.983
	Fe-O	4	1.994	0.00698	0.135			
	Fe-O	1	2.022	0.00703	0.135			
**356**	Fe-O	1	1.900	0.00743	0.99	83	6	1.982
	Fe-O	4	1.992	0.00762	0.99			
	Fe-O	1	2.020	0.00767	0.99			
**387**	Fe-O	1.3	1.892	0.00692	0.761	114	5.5	1.976
	Fe-O	1.5	1.984	0.0071	0.761			
	Fe-O	2.7	2.012	0.00715	0.761			
**420**	Fe-O	1.3	1.881	0.00696	0.38	147	5.3	1.972
	Fe-O	4	2.001	0.00719	0.38			
**452**	Fe-O	1.3	1.884	0.0079	0.36	179	5.3	1.974
	Fe-O	4	2.004	0.00816	0.36			
**484**	Fe-O	1.2	1.865	0.00736	1.216	211	5.1	1.980
	Fe-O	3.9	2.015	0.0076	1.216			
**509**	Fe-O	1.2	1.889	0.00849	1.544	236	5.1	1.981
	Fe-O	3.9	2.009	0.00877	1.544			
**538**	Fe-O	1.1	1.889	0.00878	1.632	265	4.9	1.982
	Fe-O	3.8	2.009	0.00907	1.632			

References

1. Wang, X.-S.; Ma, S.; Rauch, K.; Simmons, J. M.; Yuan, D.; Wang, X.; Yildirim, T.; Cole, W. C.; López, J. J.; Meijere, A. d.; Zhou, H.-C., Metal−Organic Frameworks Based on Double-Bond-Coupled Di-Isophthalate Linkers with High Hydrogen and Methane Uptakes. *Chem. Mater.* **2008,** *20*, 3145-3152.
2. Feng, D.; Wang, K.; Wei, Z.; Chen, Y.-P.; Simon, C. M.; Arvapally, R. K.; Martin, R. L.; Bosch, M.; Liu, T.-F.; Fordham, S.; Yuan, D.; Omary, M. A.; Haranczyk, M.; Smit, B.; Zhou, H.-C., Kinetically tuned dimensional augmentation as a versatile synthetic route towards robust metal–organic frameworks. *Nat. Commun.* **2014,** *5*, 5723.
3. Wentzcovitch, R. M.; Martins, J. L.; Price, G. D. Ab initio molecular dynamics with variable cell shape: Application to $MgSiO_3$. *Phys. Rev. Lett.* **1993**, 70, 3947–3950.
4. Cairns, A. J.; Eckert, J.; Wojtas, L.; Thommes, M.; Wallacher, D.; Georgiev, P. A.; Forster, P. M.; Belmabkhout, Y.; Ollivier, J.; Eddaoudi, M., Gaining Insights on the H2–Sorbent Interactions: Robust soc-MOF Platform as a Case Study. *Chem. Mater.* **2016,** *28* (20), 7353-7361.
